# Causal relationship between gut microbiome and childhood allergy: A bidirectional Mendelian randomization analysis

**DOI:** 10.1097/MD.0000000000047793

**Published:** 2026-02-20

**Authors:** Jiexin Zhou, Peirun Wu, Zeyu Luo, Zhuanhui Huang, Hanwei Ma, Hui Fang, Xiaoli Yin, Cuicui Yang, Ningning Sun, Yuanxiao Li

**Affiliations:** a Department of Pediatric, Fu Yang People’s Hospital, Fuyang, Anhui Province, China; b The Second Clinical Medical School, Lanzhou University, Lanzhou, Gansu Province, China; c The First Clinical Medical School, Lanzhou University, Lanzhou, Gansu Province, China; d Department of Pediatric Gastroenterology, The Second Hospital of Lanzhou University, Lanzhou, Gansu Province, China.

**Keywords:** causality, childhood allergy, gut microbiome, Mendelian randomization, metabolic pathways

## Abstract

This study aims to investigate the potential causal relationship between gut microbiome pathways and childhood allergy risk using a bidirectional Mendelian randomization (MR) approach. We conducted a bidirectional MR analysis using inverse variance weighting methods to assess the suggestive causal relationship between gut microbiome pathways and childhood allergy. The reverse MR analysis revealed that childhood allergy showed suggestive causal associations with several microbial features. For metabolic pathways, a higher risk of childhood allergy was associated with Lipid IV~A~ biosynthesis (odds ratio [OR] 1.11, 95% confidence interval [CI]: 1.00–1.22, *P* = .044) and NAD salvage pathway I (OR 1.14, 95% CI: 1.01–1.28, *P* = .030). At the taxonomic level, a lower risk of childhood allergy was associated with Bacteroidaceae (OR 0.90, 95% CI: 0.81–0.99, *P* = .031), Adlercreutzia (OR 0.79, 95% CI: 0.67–0.92, *P* = .003), Bacteroidales (OR 0.90, 95% CI: 0.81–0.99, *P* = .031), Roseburia (OR 0.89, 95% CI: 0.80–0.99,*P* = .032), Adlercreutzia equolifaciens (OR 0.79, 95% CI: 0.67–0.92, *P* = .003),Holdemania (unclassified) (OR 0.79, 95% CI: 0.64–0.98, *P* = .028), and Bacteroides vulgatus (OR 0.86, 95% CI: 0.78–0.95, *P* = .004). This study provides evidence for the potential causal relationship between specific gut microbiome pathways and childhood allergy. Several gut microbial features demonstrated suggestive causal associations with childhood allergy risk. For metabolic pathways, inverse associations (suggesting protective effects) were observed for creatinine.degradation I (OR 0.86, 95% CI: 0.76–0.98, *P* = .021) and superpathway of pyrimidine ribonucleosides degradation (OR 0.75, 95% CI: 0.62–0.91, *P* = .004). Conversely, positive associations with allergy risk were identified for isoleucine biosynthesis I from.threonine(OR 1.22, 95% CI: 1.00–1.48, *P* = .049), glutamate degradation V via hydroxyglutarate (OR 1.15, 95% CI: 1.01–1.32, *P* = .040), superpathway of polyamine biosynthesis II (OR 1.15, 95% CI: 1.01–1.32, *P* = .035), allantoin degradation to glyoxylate III (OR 1.17, 95% CI: 1.02–1.33, *P* = .024), and UDP N acetyl D glucosamine biosynthesis I (OR 1.50, 95% CI: 1.11–2.02, *P* = .008). At the taxonomic level, inverse associations were found for Odoribacter splanchnicus (OR 0.73, 95% CI: 0.58–0.91, *P* = .005) and Coprococcussp.ART55/1 (OR 0.84, 95% CI: 0.73–0.97, *P* = .014). Positive associations with allergy risk were observed for Betaproteobacteria (OR 1.29, 95% CI: 1.06–1.58, *P* = .013), Lactobacillaceae (OR 1.16, 95% CI: 1.04–1.30, *P* = .010), Clostridiaceae (OR 1.14, 95% CI: 1.01–1.29, *P* = .033), Clostridium (OR 1.15, 95% CI: 1.00–1.31, *P* = .046), Burkholderiales (OR 1.29, 95% CI: 1.06–1.58, *P* = .013), and Eubacterium hallii (OR 1.24, 95% CI: 1.08–1.41, *P* = .002).This study provides evidence for the potential causal relationship between specific gut microbiome pathways and childhood allergy.

## 1. Introduction

Since the past decade, allergic diseases have become increasingly prevalent, ranking as one of the most common non-communicable chronic diseases among children and adolescents globally.^[1]^ According to statistics, the incidence of children with food allergy has reached 8%.^[2]^ Based on the standardized International Study of Asthma and Allergies in Childhood questionnaires administered to secondary school children in Guangzhou in 1994, 2001, and 2009, the prevalence of wheezing attacks in the past year demonstrated a clear upward trend, rising from 2.0% to 5.9% and then to 7.9% over the 15-year period. The burden of allergic diseases in children has emerged as one of the primary challenges facing the healthcare system.^[3]^ Severe allergies can trigger diseases such as asthma, potentially posing life-threatening risks, and imposing a significant economic burden on families.^[4]^ Given that such diseases may be life-threatening and the prevalence is increasing, it is essential to develop effective prevention and treatment strategies.^[5]^ However, the complex mechanisms of childhood allergy, especially those related to environmental and biological factors, are still not fully understood.

Studies have shown that gut microbiota plays a key role in the occurrence and development of childhood allergy. The human gut microbiome is a complex and dynamic ecosystem, encompassing trillions of microorganisms such as bacteria, fungi, archaea, and viruses, all of which play a vital role in maintaining health.^[6]^ From the duodenum to the ileum, the intestinal microbiota has changed significantly, and the number of microorganisms has further increased, with up to 1012 bacteria in the colon.^[7]^ Actinobacteria, Firmicutes, Proteobacteria and Bacteroidetes are the 4 major bacterial genera that constitute the healthy microbiota.^[8]^ Intestinal flora plays an important role in maintaining intestinal homeostasis. The gut microbiota plays a crucial role in modulating the development and function of the host immune system. An immune microenvironment shaped by specific microbial communities can either promote health or contribute to the pathogenesis of various diseases by disrupting immune homeostasis.^[9]^ The imbalance of the composition and function of intestinal flora is likely to predict the development of food allergy.^[10]^ The mechanisms by which gut microbes regulate the immune system include the production of antimicrobial metabolites, competition with pathogenic microorganisms for nutrients, strengthening the epithelial barrier, acidifying the intestinal environment and so on.^[11]^ Dysbiosis can lead to dysregulation of the intestinal barrier, allowing allergens to enter the blood and trigger immune responses. This is believed to occur due to triggering the release of inflammatory mediators and cytokines (such as IFN-γ, TNF-α), which in turn will lead to the destruction of the intestinal barrier.^[12]^ It can be seen that changes in dietary habits will affect the composition of our microbiota, thus affecting its impact on the immune system, and then trigger allergic reactions.

This study aimed to explore the potential causal relationship between intestinal flora and childhood allergy using a 2-way MR method. Previous studies have shown that changes in gut microbiota may occur before the anaphylaxis.^[13]^ For example, a study of 141 children found that there was a significant difference in intestinal flora species between egg allergic and non allergic controls.^[14]^ In addition, some intestinal flora, such as Bifidobacterium longum and Lactobacillus plantarum, have shown anti allergic potential in both animal models and clinical trials.^[15]^ This study aims to further clarify whether the gut microbiota plays a direct role in the development of allergy in children, or whether it is only related to allergy. The results are of great significance for the development of targeted treatment and prevention strategies for childhood allergy.

## 2. Methods

### 2.1. Ethical approval

This study was approved by the Institutional Review Board of the Institutional Medical Ethics Committee of the Second Hospital of Lanzhou University approval number [2024A-1413].

### 2.2. Data sources for gut microbiome and childhood allergy

The gut microbiota-related genome-wide association studies data (GWAS) were sourced from the Dutch Microbiome Project.^[16]^ The study performed a genome-wide association study of 207 taxa and 205 pathways representing microbial composition and function within the Dutch Microbiome Project, a population cohort of 7738 individuals from the northern Netherlands. Quality-controlled genotype information was obtained for 7738 of participants for whom quality-controlled microbiome data and body mass index were also available. Of these, 58.1% were females, and ages ranged from 8 to 84 years (mean, 48.5 years). The mean body mass index value was 25.58 (range, 13.10 to 63.70). The childhood allergy-related GWAS data were sourced from FinnGen results.^[17]^ FinnGen is a large public-private partnership aiming to collect and analyze genome and health data from 500,000 Finnish biobank participants. FinnGen aims on one hand to provide novel medically and therapeutically relevant insights but also construct a world-class resource that can be applied for future studies. This study consists of 6110 cases and 406071 controls, detailed information for GWAS involved in the present MR study is shown in Table S1, Supplemental Digital Content, https://links.lww.com/MD/R422.

### 2.3. The assumptions of an MR study

**Figure 1.**
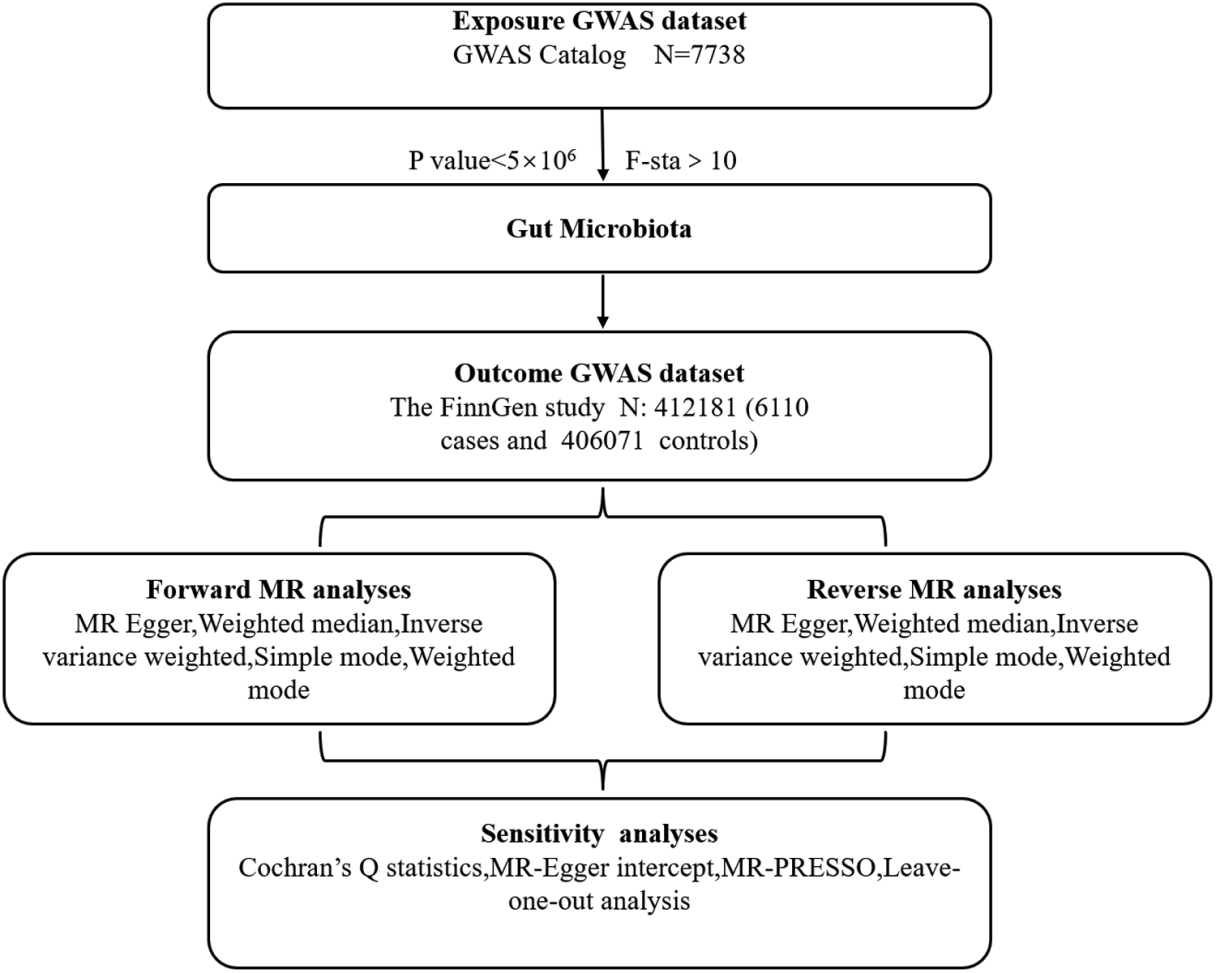
Flowchart of this study.

A valid MR study is founded on 3 fundamental assumptions, as illustrated in Figure 1.^[18]^ First, the genetic variants selected as instrumental variables (IVs) must exhibit a strong association with the exposure of interest. The strength of these instruments is typically assessed using the F statistic, which can be computed using the formula *F* = *R*^2^(n-k-1)/*k*(1-*R*^2^), where *R*^2^denotes the proportion of variance explained by the instruments, n indicates the sample size, and *k* represents the number of selected IVs. For the purposes of this study, we adopt a conventional threshold of an F statistic >10 to mitigate the risk of weak instrument bias.^[19]^

Second, it is essential that there are no unmeasured confounders influencing the relationship between the genetic variants and the outcome. Third, the genetic variants must influence the outcome solely through their effect on the exposure of interest, thereby ensuring the absence of horizontal pleiotropy between the genetic variants and the outcome. Genome-wide significant and independent single-nucleotide polymorphisms (SNPs) that were used as instruments for gut microbiota is shown in Table S2, Supplemental Digital Content, https://links.lww.com/MD/R422. Genome-wide significant and independent SNPs that were used as instruments for childhood allergy is shown in Table S3, Supplemental Digital Content, https://links.lww.com/MD/R422.

### 2.4. Mendelian randomization analysis

We estimated the causal relationships between 207 taxa and 205 pathways and childhood asthma separately using different MR methods. A 2-sided *P*-value that passed the Bonferroni correction *P*-value was defined as statistically significant, which is 1.21 × 10 − 4 (0.05/412) for gut microbiota. A *P* < .05, but above the Bonferroni-corrected threshold, was considered as suggestive for association. Reverse MR analysis was employed to confirm the causal direction. It followed similar methods as forward MR. *P* < .05 was defined as statistically significant. To detect the causal relationships between exposure and outcomes, we conducted an MR analysis employing 4 distinct methodologies, MR-Egger, weighted median, random-effect inverse variance weighted (IVW) and weighted mode. The MR-Egger approach provides a consistent estimate of causal effects, even in the presence of pleiotropic effects from all genetic variants, provided that the association between each genetic variant and the exposure remains independent of these pleiotropic influences.^[20]^ The weighted median method necessitates that a minimum of 50% of the analytical weight is derived from genetic variants that are considered valid instruments.^[21]^ The IVW method functions as a meta-analytic technique predicated on the assumption that the instruments influence the outcome solely through the exposure of interest, without any alternative pathways.^[22]^ The weighted mode approach is deemed consistent when the largest subset of instruments that identify the same causal effect are valid, even if the majority of the remaining instruments are invalid.^[23]^ Consequently, a suggestive causal relationship was inferred when a statistically significant *P* value (*P* < .05) was observed from any of the IVW methodologies employed in the MR analysis.

A sensitivity analysis was conducted on significant estimates to identify potential heterogeneity and pleiotropy. The Cochran *Q* test was employed to detect heterogeneity, while the MR-Egger intercept test was utilized to evaluate horizontal pleiotropy. A *P* value >.05 (*P* > .05) was interpreted as indicative of the absence of heterogeneity or pleiotropy. Furthermore, leave-one-out analyses were performed to determine whether the causal estimates were influenced or biased by any single SNP. This was achieved by sequentially removing each instrumental SNP and re-executing the IVW analyses.

All analyses were conducted using R (version 4.2.1), employing the R packages “TwoSampleMR” (version 0.5.6) and “stats” (version 4.2.1).

## 3. Result

### 3.1. Causal relationship between gut microbiome and childhood allergy

**Figure 2.**
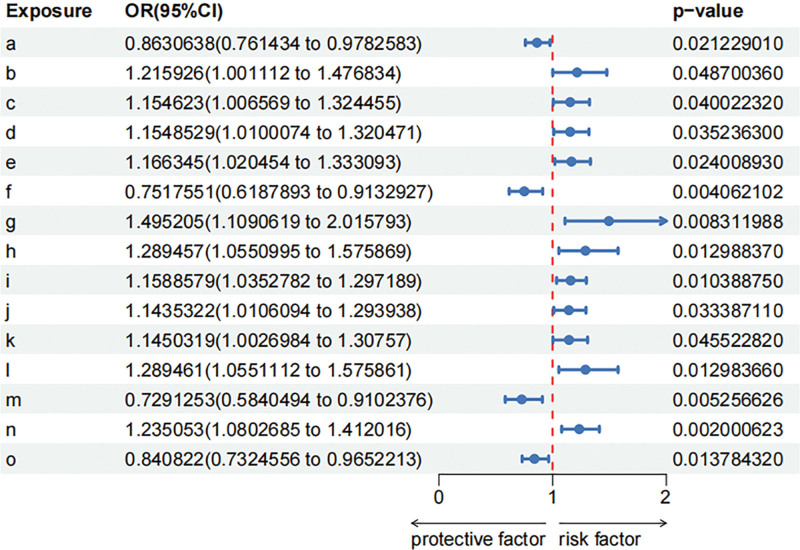
Gut microbiome which associated with childhood allergy.

This MR analysis investigated causal associations between specific gut microbiota pathways and the risk of childhood allergy using IVW. The findings reveal several gut microbiome pathways and taxa significantly associated with childhood allergy risk, supported by supplementary analyses for heterogeneity and pleiotropy. MR results from gut microbiome to childhood allergy in discovery dataset is shown in Figure S1, Supplemental Digital Content, https://links.lww.com/MD/R423, Table S4, Supplemental Digital Content, https://links.lww.com/MD/R422. This MR analysis investigated causal associations between specific gut microbiota pathways and the risk of childhood allergy using IVW. The findings reveal several gut microbiome pathways and taxa significantly associated with childhood allergy risk, supported by supplementary analyses for heterogeneity and pleiotropy. MR results from gut microbiome to childhood allergy in discovery dataset is shown in Figure S1, Supplemental Digital Content, https://links.lww.com/MD/R423, Table S4, Supplemental Digital Content, https://links.lww.com/MD/R422. For metabolic pathways, we found a suggestive causal association of increase in creatinine.degradation I (OR 0.86, 95% CI: 0.76–0.98, *P* = .021), superpathway of pyrimidine ribonucleosides degradation (OR 0.75, 95% CI: 0.62–0.91, *P* = .004) and lower risk of childhood allergy. we found a suggestive causal association of increase in isoleucine biosynthesis I from.threonine (OR 1.22,95% CI: 1.00–1.48, *P* = .049), glutamate degradation V via hydroxyglutarate (OR 1.15,95% CI: 1.01–1.32,*P* = .040), superpathway of polyamine biosynthesis II (OR 1.15, 95% CI: 1.01–1.32, *P* = .035),allantoin degradation to glyoxylate III (OR 1.17, 95% CI: 1.02–1.33, *P* = .024),UDP N acetyl D glucosamine biosynthesis I (OR 1.50, 95% CI: 1.11–2.02, *P* = .008) and higher risk of childhood allergy. At the taxonomic level, we found a suggestive causal association of increase in Odoribacter_splanchnicus (OR 0.73, 95% CI: 0.58–0.91, *P* = .005), Coprococcus sp.ART55/1 (OR 0.84, 95% CI: 0.73–0.97, *P* = .014) and lower risk of childhood allergy. we found a suggestive causal association of increase in Betaproteobacteria (OR 1.29, 95% CI: 1.06–1.58, *P* = .013), Lactobacillaceae (OR 1.16, 95% CI: 1.04–1.30, *P* = .01), Clostridiaceae (OR 1.14, 95% CI: 1.01–1.29, *P* = .033), Clostridium (OR 1.15, 95% CI: 1.0–1.31, *P* = .046), Burkholderiales (OR 1.29, 95% CI: 1.06–1.58, *P* = .013), Eubacterium_hallii (OR 1.24, 95% CI: 1.08–1.41, *P* = .002) and higher risk of childhood allergy. IVs exhibited homogeneity (Cochran *Q*: *P* > .05). No evidence of horizontal pleiotropy was detected (MR-Egger intercept and MR-PRESSO: *P* > .05). Sensitivity analysis of the causal association between gut microbiome and childhood allergy is shown in Figure S2, Supplemental Digital Content, https://links.lww.com/MD/R423, Table S6, Supplemental Digital Content, https://links.lww.com/MD/R422.. IVs exhibited homogeneity (Cochran *Q*: *P* > .05). No evidence of horizontal pleiotropy was detected (MR-Egger intercept and MR-PRESSO: *P* > .05), the forward forest plot is shown in Figure 2.

(A) Creatinine.degradation I; (B) Isoleucine biosynthesis I from.threonine; (C) Glutamate degradation V via hydroxyglutarate; (D) Superpathway of polyamine biosynthesis II; (E) Allantoin degradation to glyoxylate III; (F) Superpathway of pyrimidine ribonucleosides degradation; (G) UDP N acetyl D glucosamine biosynthesis I; (H) Betaproteobacteria; (I) Lactobacillaceae; (J) Clostridiaceae; (K) Clostridium; (L) Burkholderiales; (M) Odoribacter_splanchnicus; (N) Eubacterium_hallii; (O) Coprococcus_sp_ART55_1.

### 3.2. Causal relationship between childhood allergy and gut microbiome

**Figure 3.**
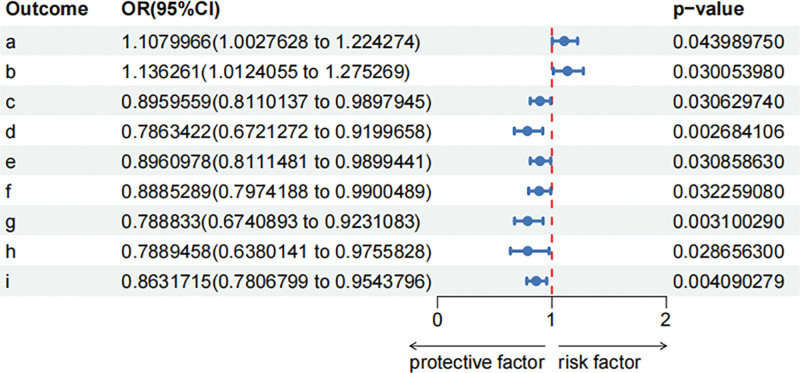
Childhood allergy which associated with gut microbiome.

In this MR analysis exploring the bidirectional relationship between childhood allergy and gut microbiota, we identified several statistically significant associations across bacterial pathways and genera. Key findings include both positive and inverse associations, suggesting differential effects depending on microbial pathways and taxa. MR results from childhood allergy to gut microbiome in discovery dataset is shown in Figure S3, Supplemental Digital Content, https://links.lww.com/MD/R423, Table S5, Supplemental Digital Content, https://links.lww.com/MD/R422. In this MR analysis exploring the bidirectional relationship between childhood allergy and microbiome, we identified several statistically significant associations across bacterial pathways and genera. Key findings include both positive and inverse associations, suggesting differential effects depending on microbial pathways and taxa. MR results from childhood allergy to gut microbiome in discovery dataset is shown in Figure S3, Supplemental Digital Content, https://links.lww.com/MD/R423, Table S5, Supplemental Digital Content, https://links.lww.com/MD/R422. For metabolic pathways, we found a suggestive causal association of childhood allergy and higher in Lipid IV~A~ biosynthesis (OR1.11, 95% CI:1.0–1.22, *P* = .044), NAD salvage pathway I (OR 1.14, 95% CI: 1.01–1.28, *P* = .030). At the taxonomic level, we found a suggestive causal association of childhood allergy and lower in Bacteroidaceae (OR 0.90, 95% CI: 0.81–0.99, *P* = .031), Adlercreutzia (OR 0.79, 95% CI: 0.67–0.92, *P* = .003), Bacteroidales (OR 0.90, 95% CI: 0.81–0.99, *P* = .031), Roseburia (OR 0.89, 95% CI: 0.80–0.99, *P* = .032), Adlercreutzia equolifaciens (OR 0.79, 95% CI: 0.67–0.92, *P* = .003), Holdemania unclassified (OR 0.79, 95% CI: 0.64–0.98, *P* = .028), Bacteroides_ vulgatus (OR 0.86, 95% CI: 0.78–0.95, *P* = .004), the reverse forest plot is shown in Figure 3. Sensitivity analysis of the causal association between childhood allergy and gut microbiome is shown in Figure 4, Supplemental Digital Content, https://links.lww.com/MD/R423, Supplementary Table S7, Supplemental Digital Content, https://links.lww.com/MD/R422, IVs exhibited homogeneity (Cochran *Q*: *P* > .05). No evidence of horizontal pleiotropy was detected (MR-Egger intercept and MR-PRESSO: *P* > .05)

(A) Lipid IV~A~ biosynthesis; (B) NAD salvage pathway I; (C) Bacteroidaceae; (D) Adlercreutzia; (E) Bacteroides; (F) Roseburia; (G) Adlercreutzia_equolifaciens; (H) Holdemania_unclassified; (I) Bacteroides_vulgatus.

## 4. Discussion

Growing evidence shows a significant association between intestinal flora and allergic diseases, specifically atopic dermatitis, allergic rhinitis, and allergic asthma.^[24]^ The microflora hypothesis suggests that gut microbial dysbiosis in early life might trigger hypersensitivity disorders.^[25]^ Though hygiene hypothesis has been the major hypothesis to explain epidemiological phenomena, it is also applied to explain the trends in autoimmune diseases, such as type 1 diabetes mellitus, multiple sclerosis,^[26–28]^ and chronic inflammatory diseases, including inflammatory bowel disease and neuro-inflammatory disorder^.[29]^ However, the association analysis has not yet been clarified on the relationship between gut microbiota species and their metabolic pathways to allergy risk in children. Based on the results that repeated exposures to infection in early life after birth led to fewer incidences of hypersensitive immune diseases, Strachan^[30]^ proposed the hygiene hypothesis in 1989.

Our study reveals significant associations between specific metabolic pathways and bacterial taxa with childhood allergy risk, demonstrating both protective and risk-increasing effects across different microbial metabolic functions. We also discovered that there exist some potential causal relationships between childhood allergy and specific gut microbiome pathways and taxa. After identifying which gut microbial pathways that were significantly associated with the risk of allergies in children, we performed sensitivity analyses in order to explore Is there significant heterogeneity and horizontal pleiotropy present.^[31,32]^We discovered that there exist some potential causal relationships between specific gut microbiome pathways and taxa and childhood allergy. Recent studies indicate that Odoribacter splanchnicus and its derived extracellular vesicles possess therapeutic potential for inflammatory bowel disease, potentially through mechanisms involving the regulation of intestinal inflammation and barrier function. These activities may consequently contribute to a reduced risk of childhood allergies.^[33]^Within the human gut microbiota, the genus Coprococcus (phylum Firmicutes) contributes to immune regulation through its role in the production of short-chain fatty acids, such as butyrate. Butyrate is known to influence T-cell activity, which promotes intestinal immune tolerance, suggesting a mechanism by which Coprococcus may help lower susceptibility to allergic diseases.^[34]^ Accumulating clinical evidence supports that specific probiotic strains, notably Lactobacillus rhamnosusGG, can assist in treating cow’s milk protein allergy by promoting tolerance acquisition and alleviating gastrointestinal and other allergic symptoms.^[35]^ This observed clinical benefit appears to contradict our MR findings, which did not detect a protective causal effect. This discrepancy may arise from confounding factors inherent in the MR design. Research indicates that gut microbiota dysbiosis, particularly an elevated relative abundance of Clostridium, may be a contributing factor in the development and progression of allergic diseases.^[36]^A study revealed that the gut microbiota of children with food protein-induced enterocolitis syndrome is enriched with taxa and genes associated with *Escherichia coli*. As a member of the Enterobacteriaceae family, Burkholderiales may contribute to the pathogenesis of food protein-induced enterocolitis syndrome.^[37]^ Direct evidence investigating the specific association between Betaproteobacteria, Eubacterium hallii and childhood allergy is currently limited in the available literature.

We also discovered that there exist some potential causal relationships between childhood allergy and specific gut microbiome pathways and taxa. A consistent body of research indicates that gut microbial dysbiosis is a feature of food allergy in children, characterized by a significantly lower relative abundance of Bacteroidaceae compared to the gut microbiota of healthy children.^[38]^ A study revealed that the abundance of Bacteroidales in the gut of children with food allergies was significantly lower than in healthy controls.^[39]^ Compared to healthy children, allergic children showed a reduced intestinal abundance of Roseburia, a butyrate-producing bacterium known to have immunomodulatory effects, which may underlie its potential role in mitigating allergic responses.^[36]^ Direct evidence investigating the specific association between childhood allergy and Adlercreutzia, Adlercreutzia equolifaciens, Holdemania unclassified, Bacteroides_ vulgatus is currently limited in the available literature.

This study has some limitations. First, our analysis focused exclusively on European populations. Second, while IVW estimates reached statistical significance, MR-Egger and weighted mode analyses did not. Though concordance across all methods is ideal, IVW remains the most statistically robust MR approach, particularly when compared to MR-Egger. Thirdly, several gut microbiota features demonstrated statistical significance (e.g., *P*-value < .05) in their association with childhood allergy, their effect sizes were modest (e.g., odds ratios close to 1), indicating findings of marginal significance. Therefore, the biological relevance of these associations should be interpreted with caution. While MR implies a potential causal link, the limited magnitude of these effects suggests that individual microbial features may have only a modest direct influence on childhood allergy risk at the population level.

This MR study demonstrates a potential causal relationship between GM and childhood allergy. The specific bacterial composition identified may modulate allergy development in children, highlighting potential targets for prevention and therapeutic intervention.

## Acknowledgments

The FinnGen study is a large-scale genomics initiative that has analyzed over 500,000 Finnish biobank samples and correlated genetic variation with health data to understand disease mechanisms and predispositions. The project is a collaboration between research organizations and biobanks within Finland and international industry partners. This research was funded by the Cuiying Scientific Training Program for Undergraduates of Lanzhou University Second Hospital (CYXZPT2025-36), Student Entrepreneurship and Innovation Action Plan of Lanzhou University (20240050147 and 20250050021), Taishun County Science and Technology Plan Project(2025-TSXM0017), and Guided Science and Technology Plan Project of Chengguan District, Lanzhou: Social Development Program(2025-zdx-2).

## Author contributions

**Conceptualization:** Xiaoli Yin, Yuanxiao Li.

**Funding acquisition:** Yuanxiao Li.

**Formal analysis:** Jiexin Zhou, Peirun Wu, Zeyu Luo, Zhuanhui Huang.

**Writing – original draft:** Hanwei Ma, Hui Fang, Xiaoli Yin, Cuicui Yang, Ningning Sun, Yuanxiao Li.

## Supplementary Material
