# Supplementary material for: Causal relationship between gut microbiome and childhood allergy: A bidirectional Mendelian randomization analysis
Source: Medicine (Baltimore). 2026 Feb 20;105(8):e47793. doi: 10.1097/MD.0000000000047793 (PMC12928942; doi:10.1097/MD.0000000000047793)

Figure S1. Scatter plots for the causal relationship between gut microbiome and childhood allergy in forward MR analyses.

CRNFORCAT.PWY..creatinine.degradation.I FERMENTATION.PWY..mixed.acid.fermentation


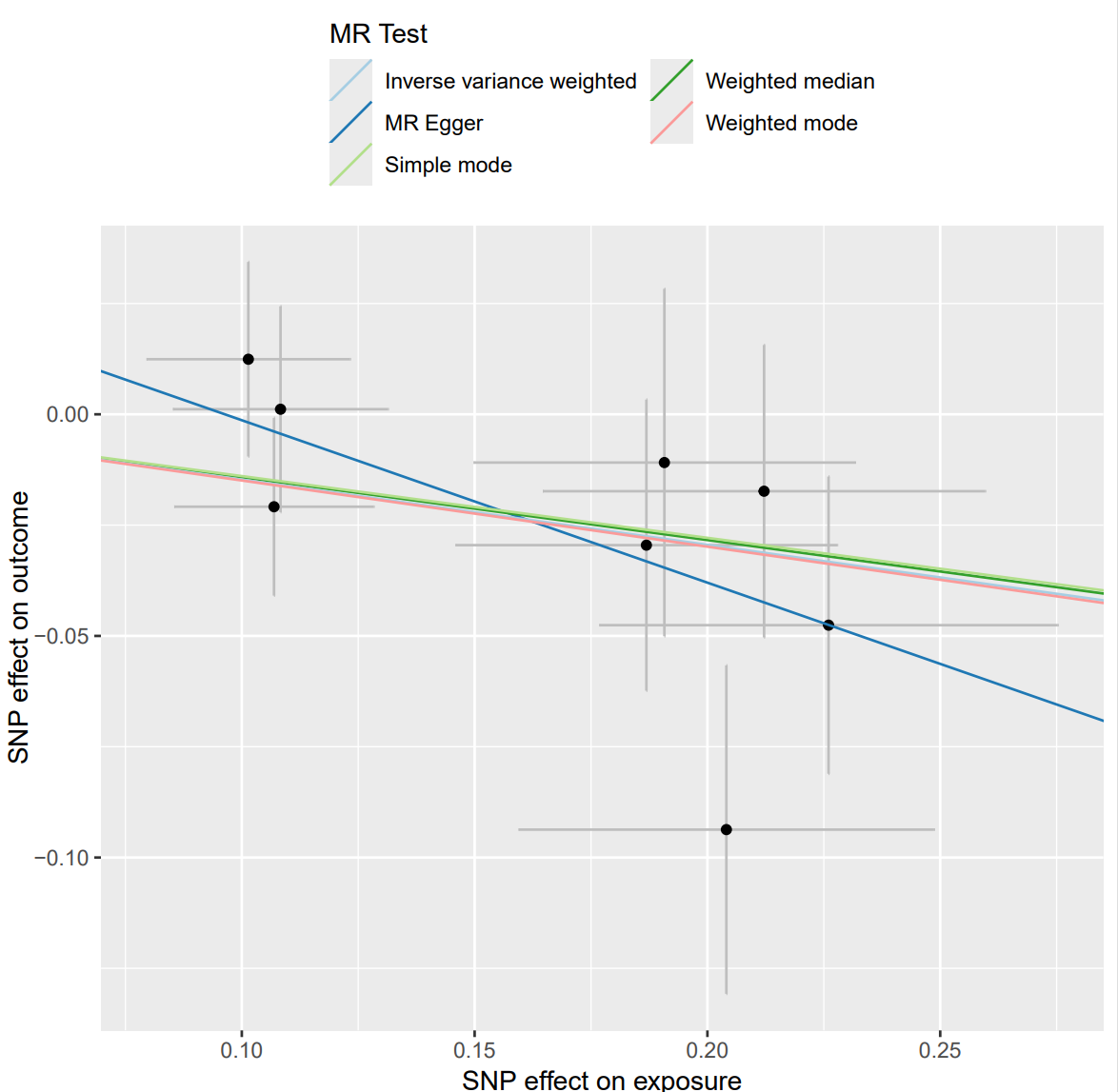

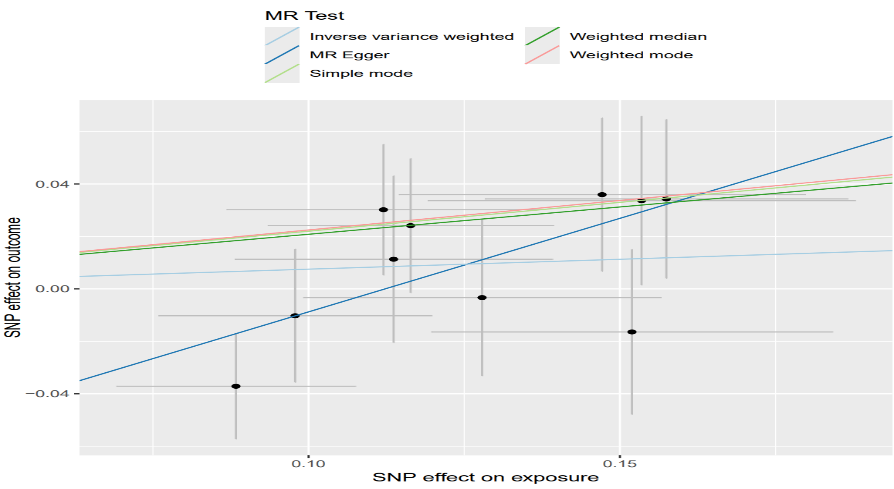


ILEUSYN.PWY..L.isoleucine.biosynthesis.I..from.threonine. P162.PWY..L.glutamate.degradation.V..via.hydroxyglutarate.
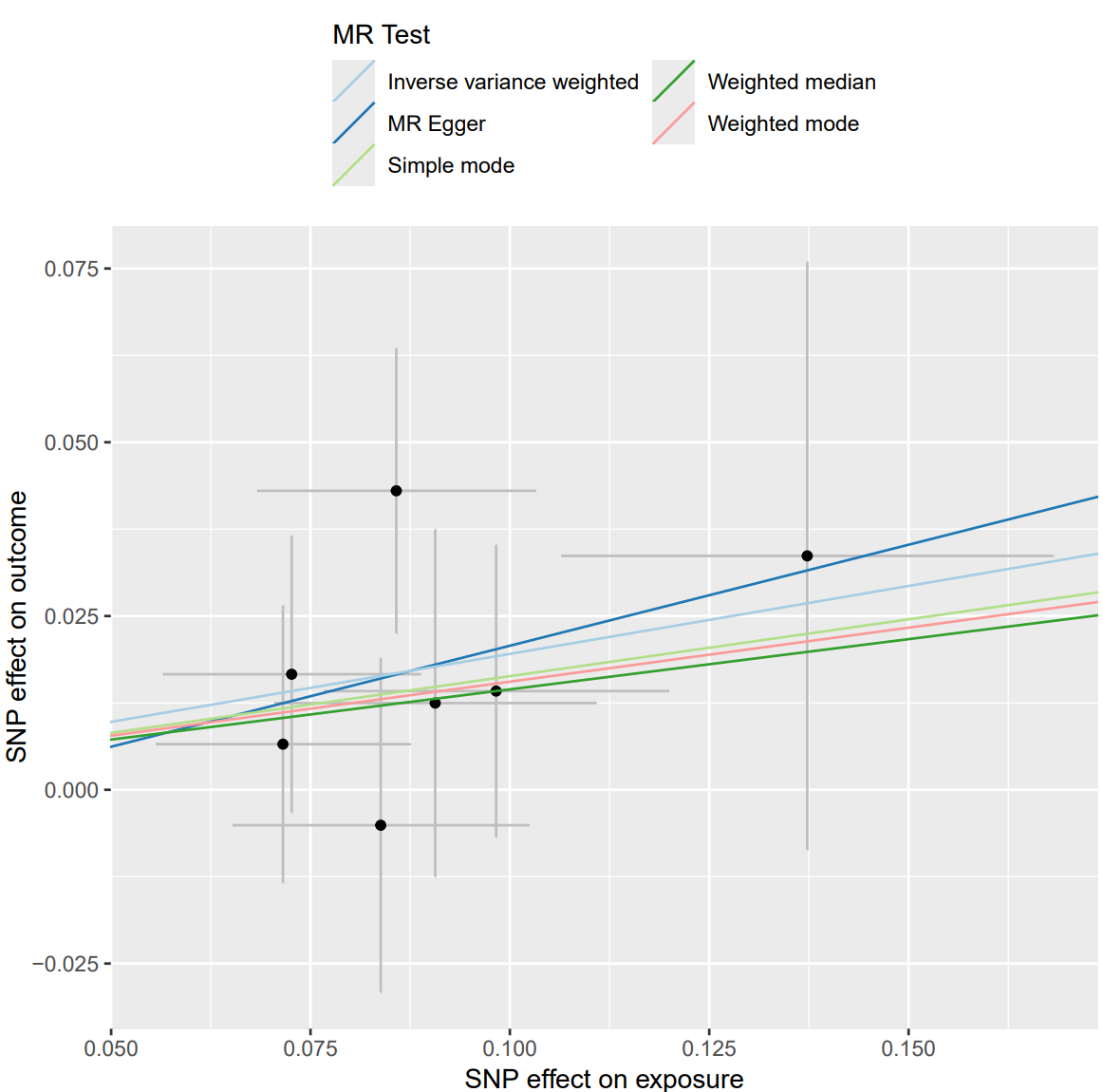

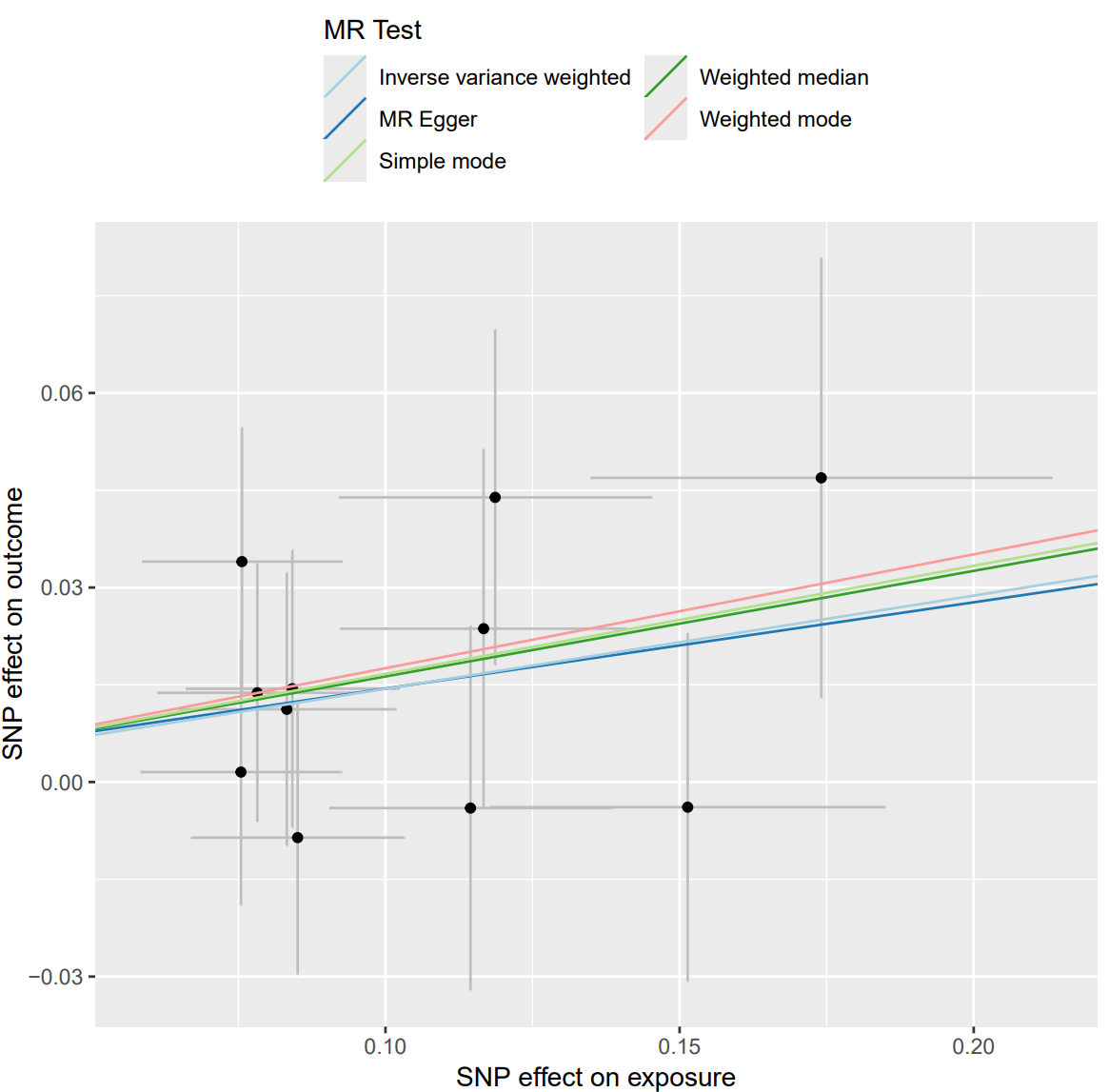


POLYAMINSYN3.PWY..superpathway.of.polyamine.biosynthesis.II PWY.5705..allantoin.degradation.to.glyoxylate.III


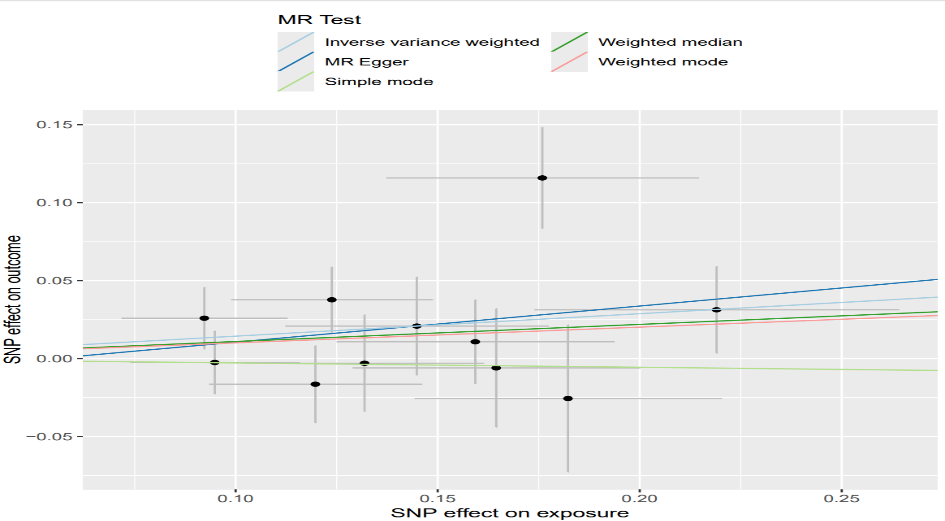

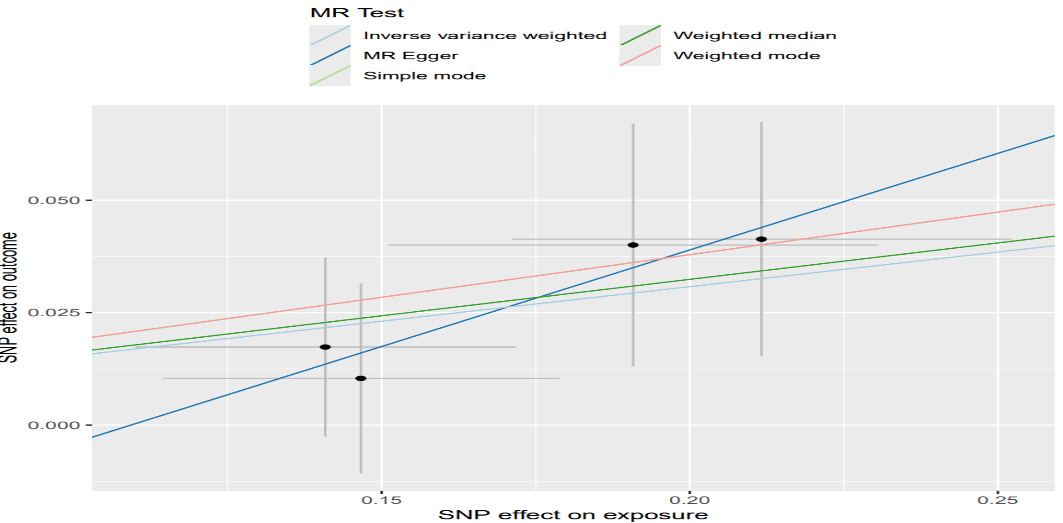


PWY.5791..1.4.dihydroxy.2.naphthoate.biosynthesis.II..plants. PWY.7209..superpathway.of.pyrimidine.ribonucleosides.degradation


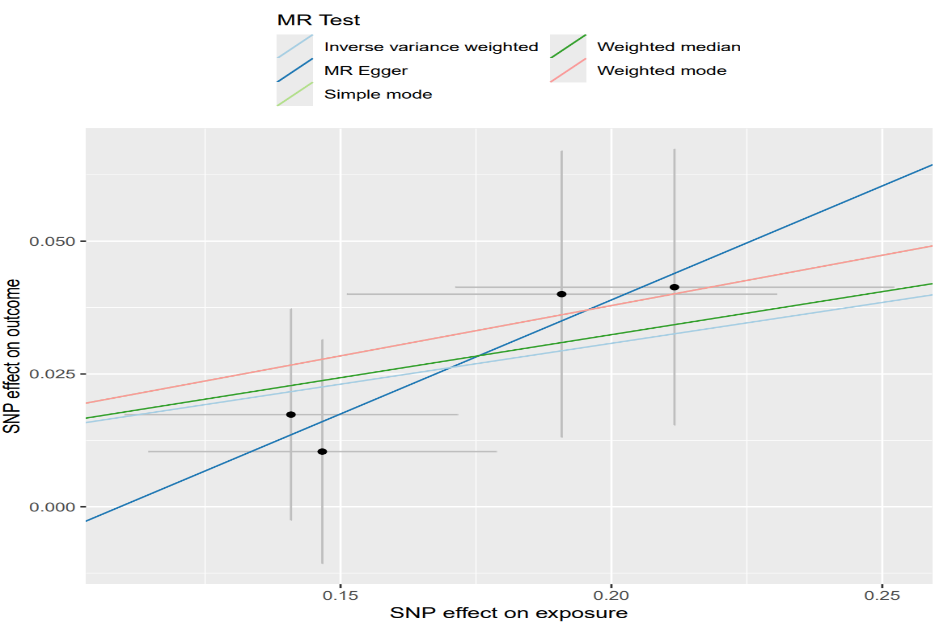

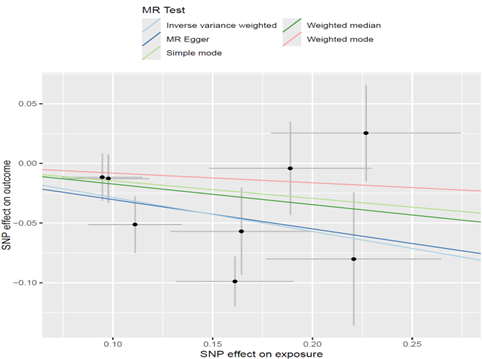


UDPNAGSYN.PWY..UDP.N.acetyl.D.glucosamine.biosynthesis.I k_Bacteria.p__Proteobacteria.c__Betaproteobacteria


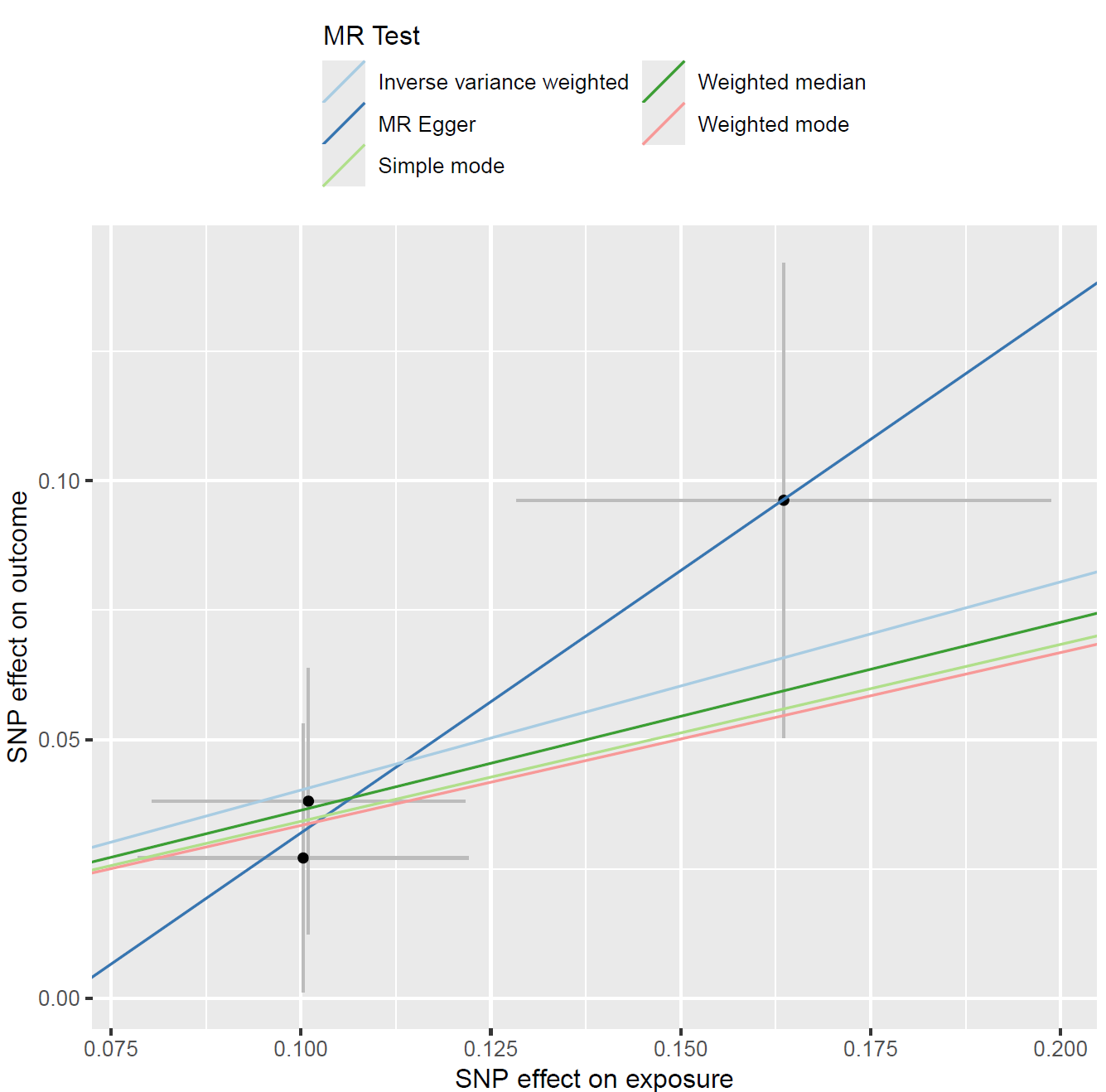

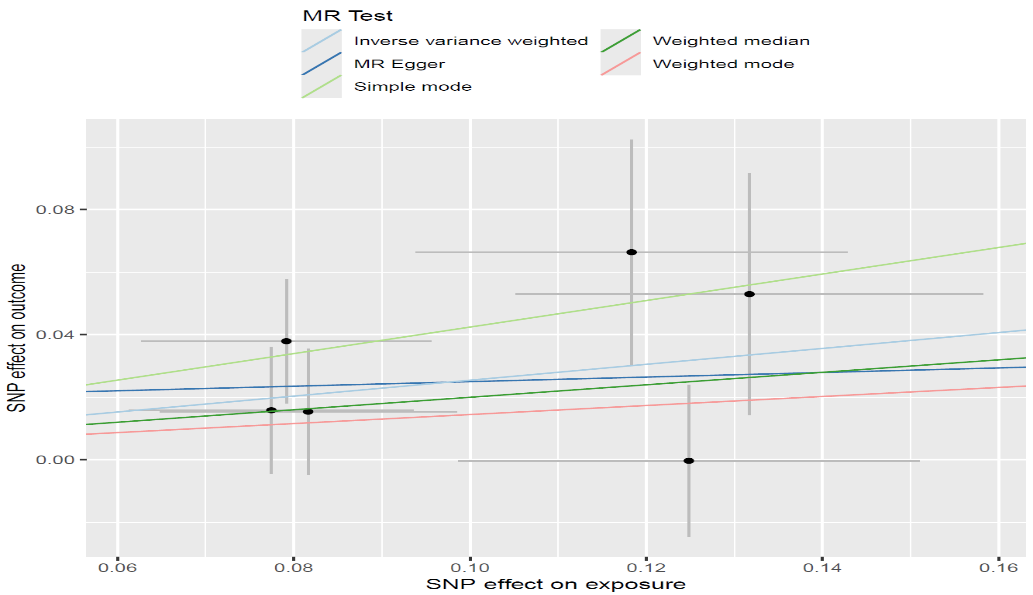


k__Bacteria.p__Firmicutes.c__Bacilli.o__Lactobacillales.f__Lactobacillaceae k__Bacteria.p__Firmicutes.c__Clostridia.o__Clostridiales.f__Clostridiaceae


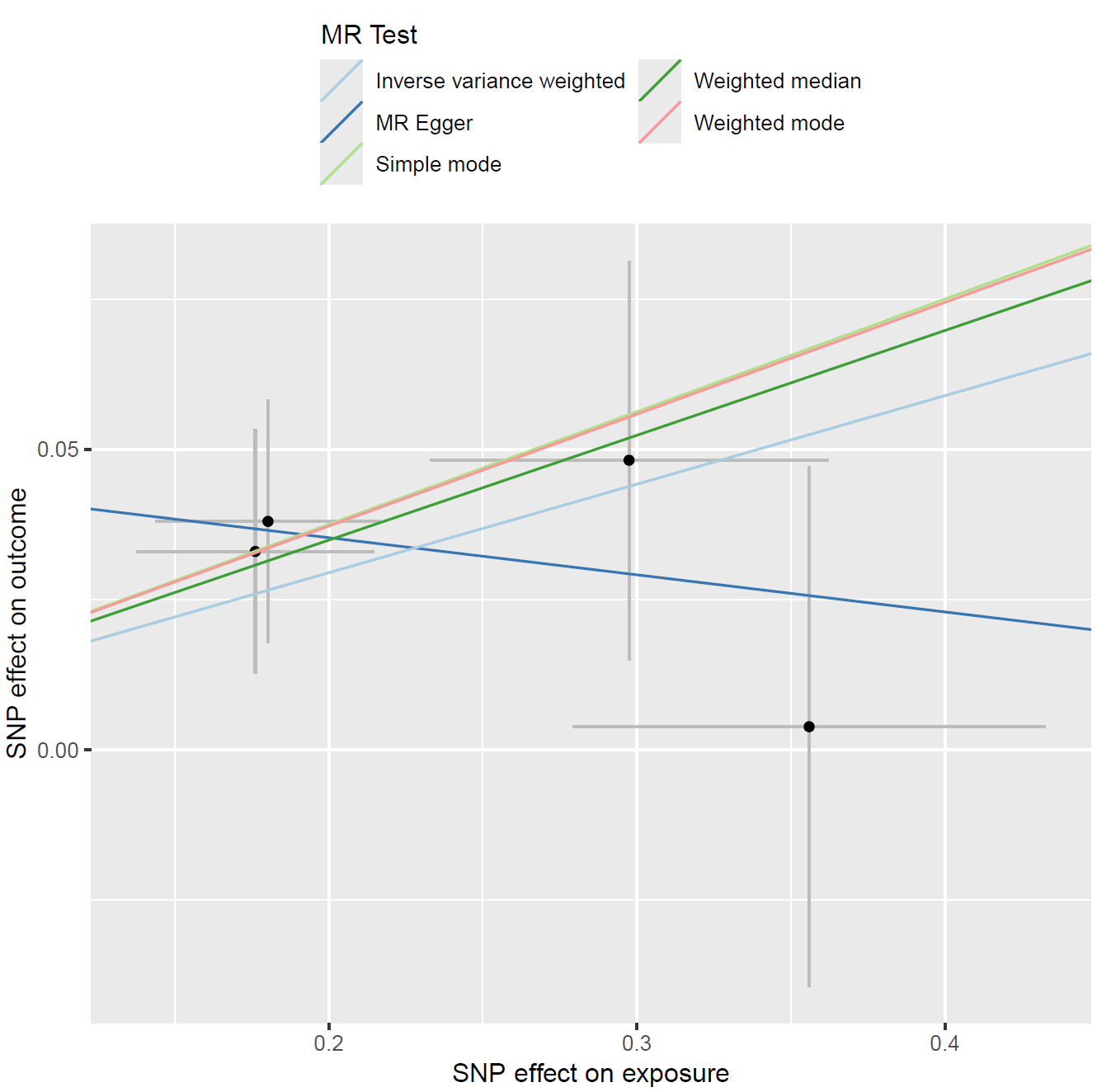

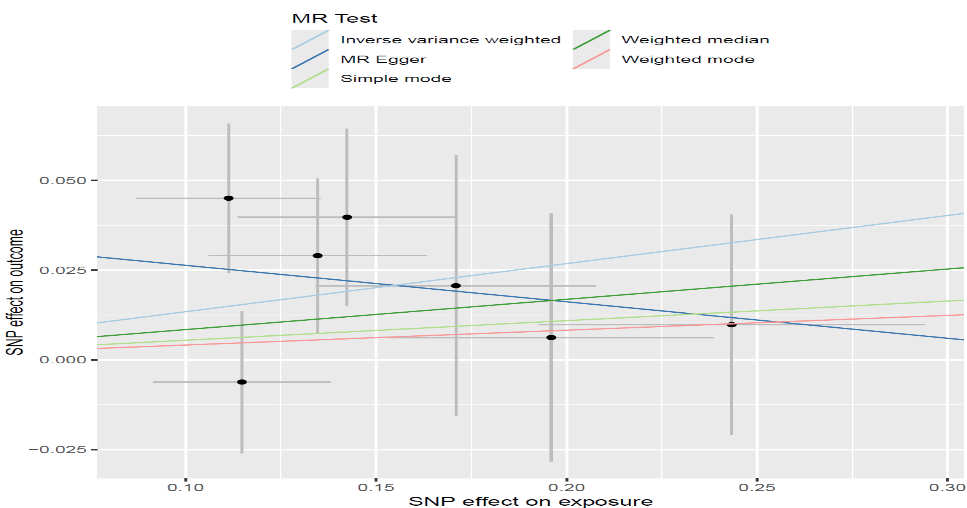


k__Bacteria.p__Firmicutes.c__Clostridia.o__Clostridiales.f__Clostridiaceae.g__Clostridium k__Baceria.p__Proteobacteria.c__Betaproteobacteria.o__Burkholderiales


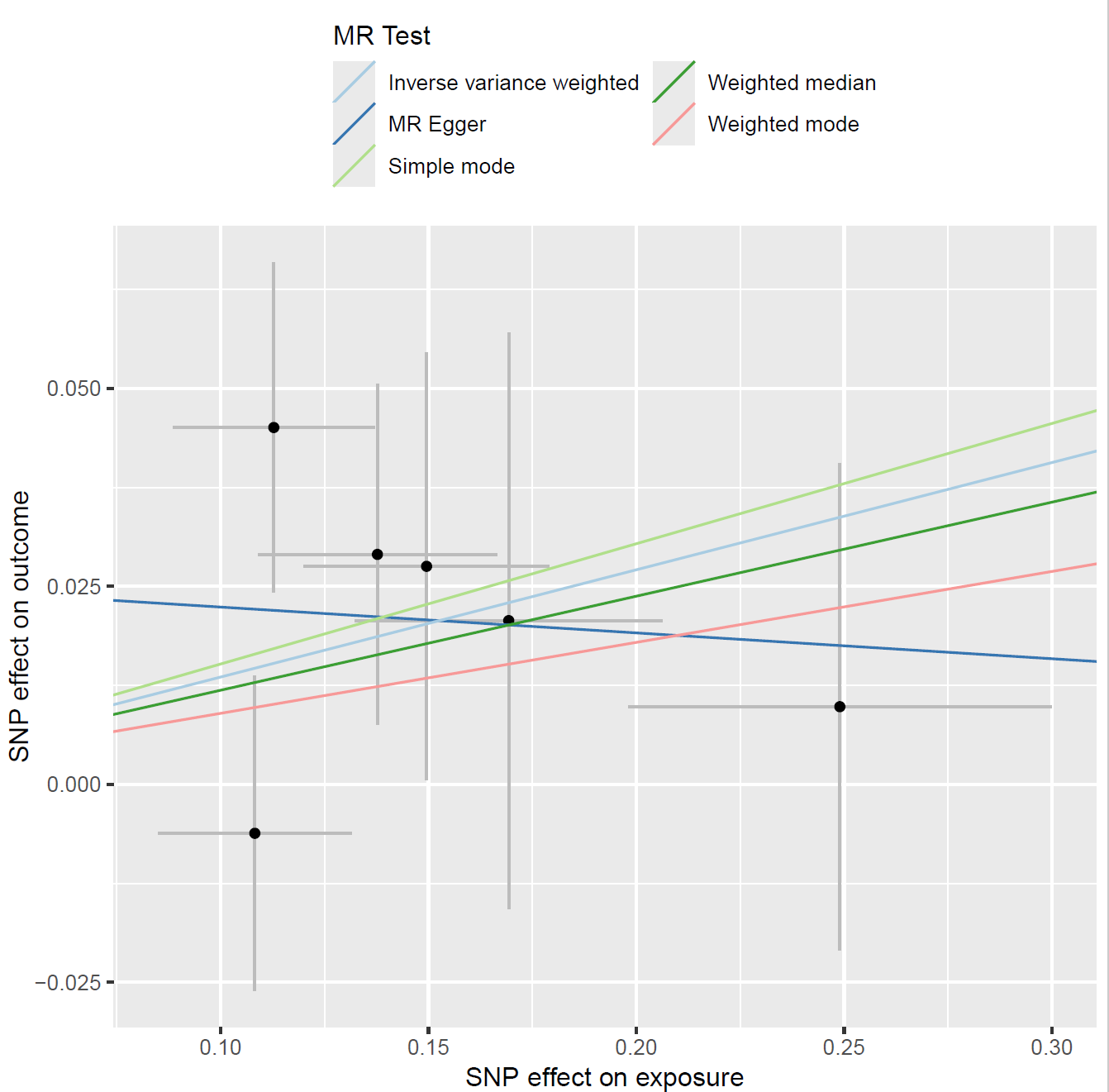

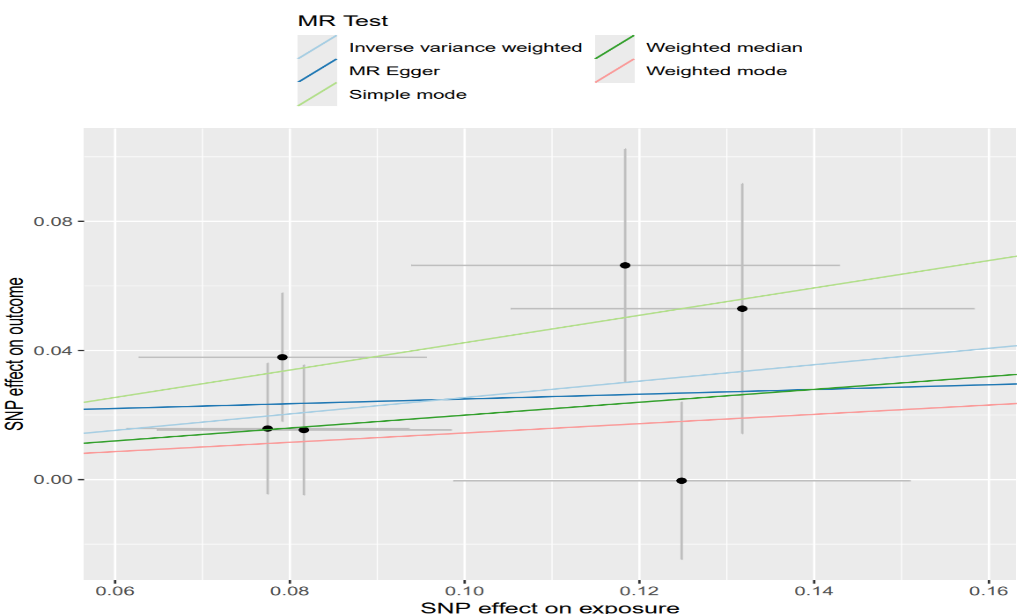


k__Bacteria.p__Bacteroidetes.c__Bacteroidia.o__Bacteroidales.f__Porphyromonadaceae.g__Odoribacter.s__Odoribacter_splanchnicus


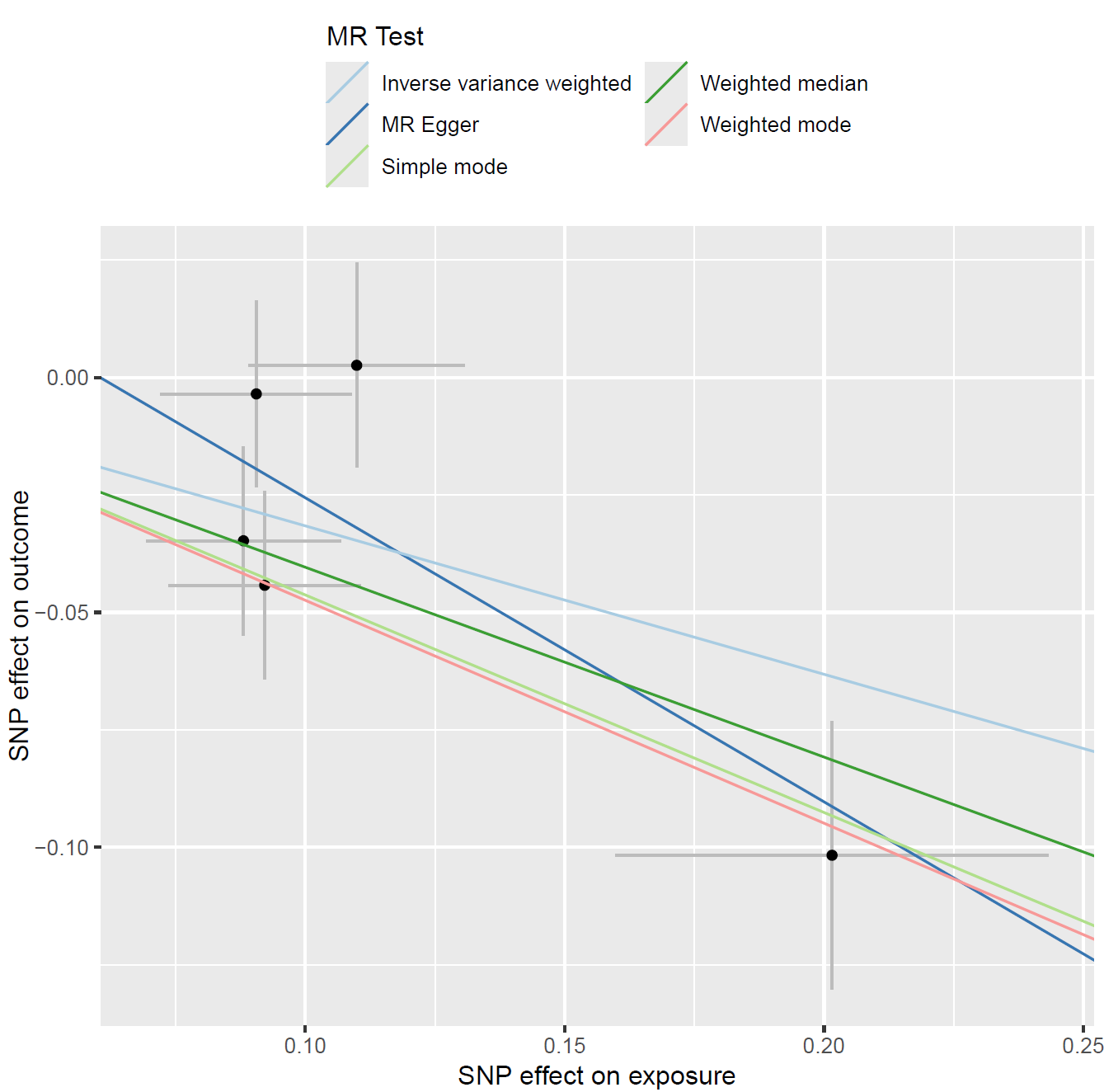


k__Bacteria.p__Firmicutes.c__Clostridia.o__Clostridiales.f__Eubacteriaceae.g__Eubacterium.s__Eubacterium_hallii


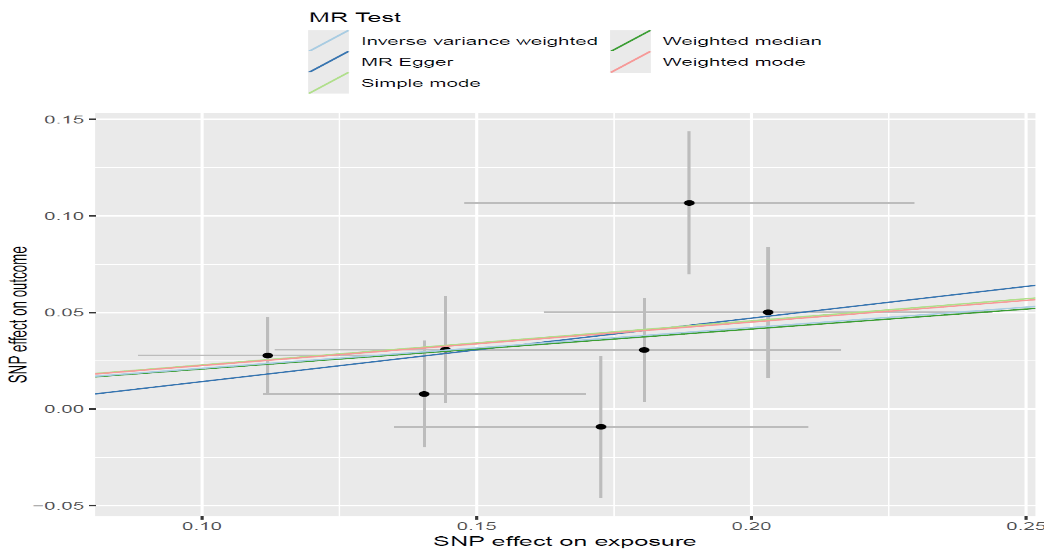


k__Bacteria.p__Firmicutes.c__Clostridia.o__Clostridiales.f__Lachnospiraceae.g__Coprococcus.s__Coprococcus_sp_ART55_1


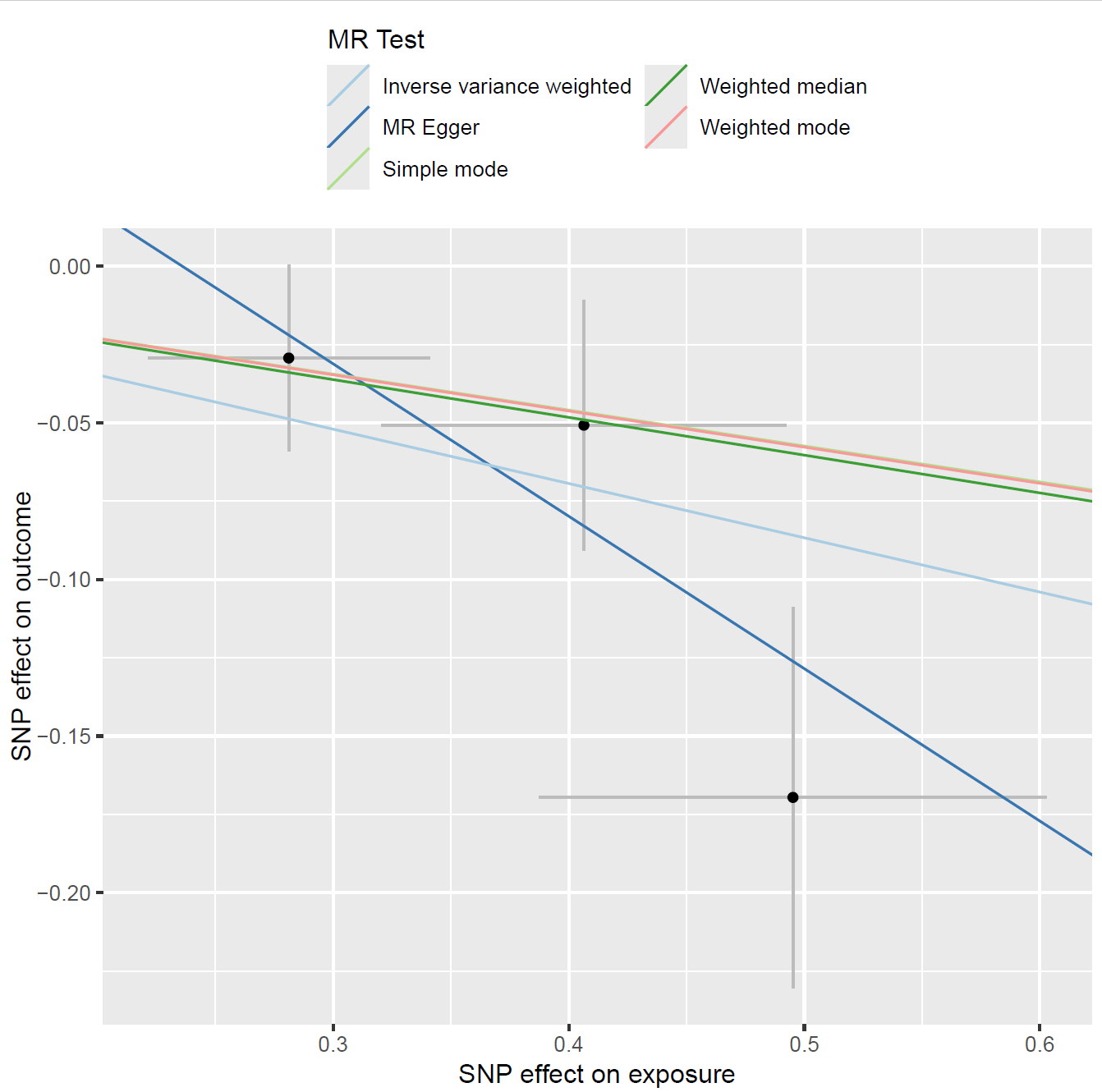


Figure S2. Leave-one-out plots for the causal association between gut microbiome and childhood allergy in forward MR analyses.

CRNFORCAT.PWY..creatinine.degradation.I FERMENTATION.PWY..mixed.acid.fermentation


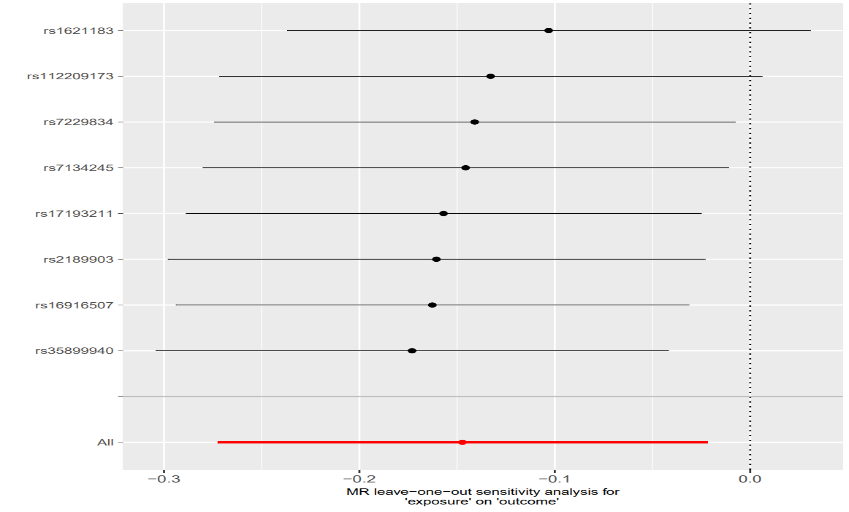

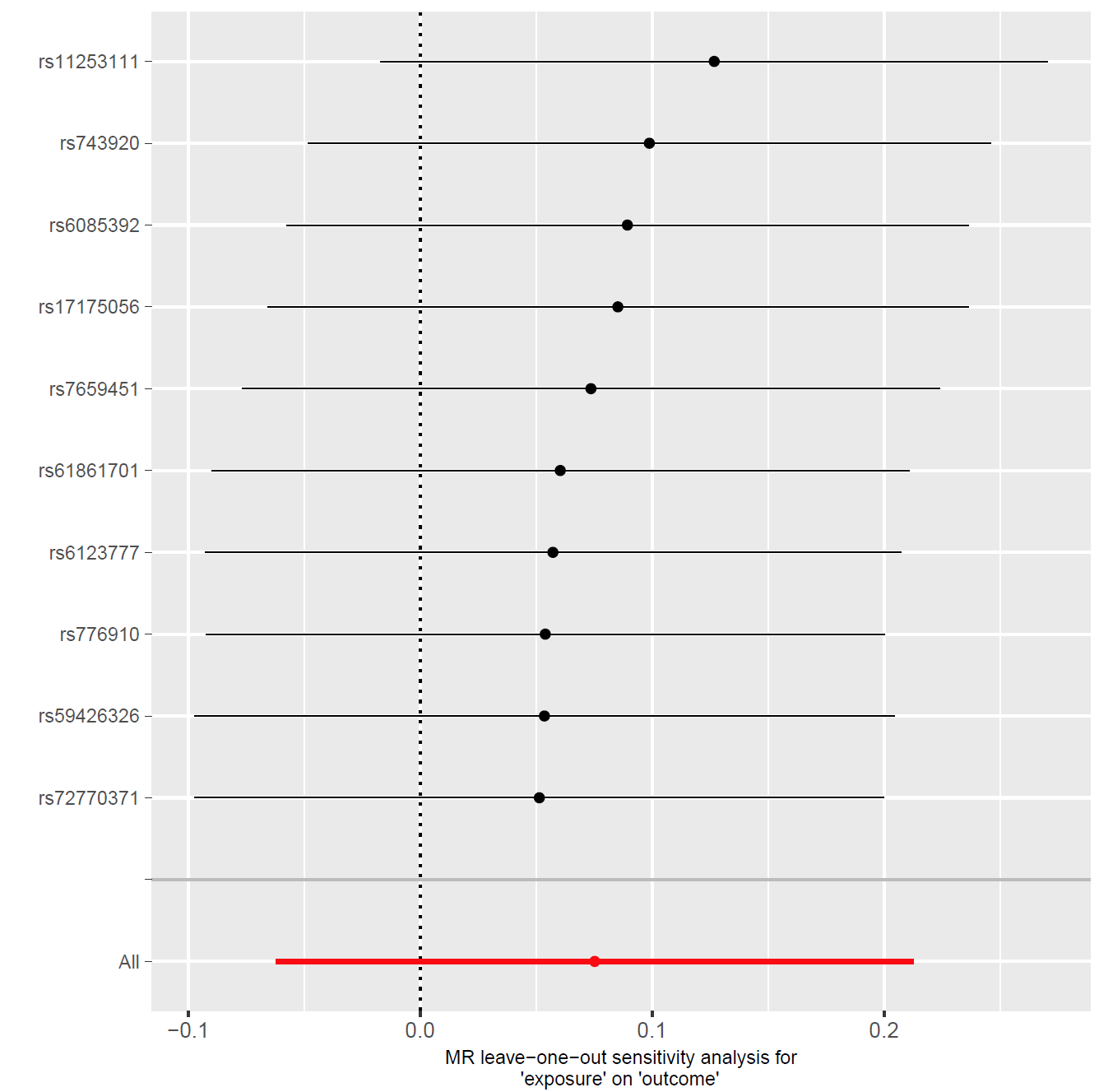


ILEUSYN.PWY..L.isoleucine.biosynthesis.I..from.threonine. P162.PWY..L.glutamate.degradation.V..via.hydroxyglutarate.


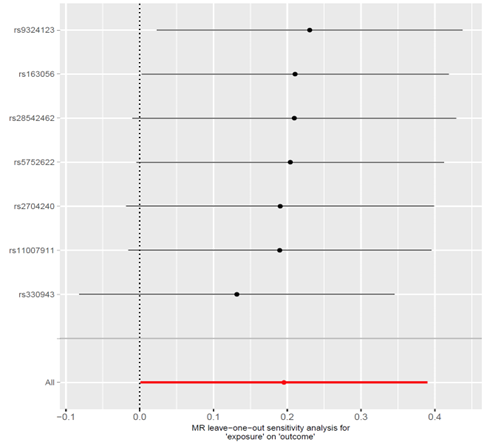

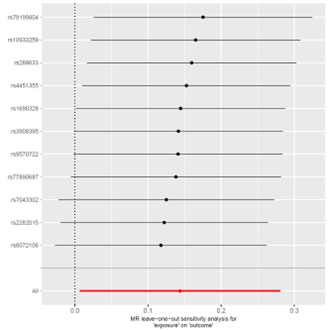


POLYAMINSYN3.PWY..superpathway.of.polyamine.biosynthesis.II PWY.5705..allantoin.degradation.to.glyoxylate.III


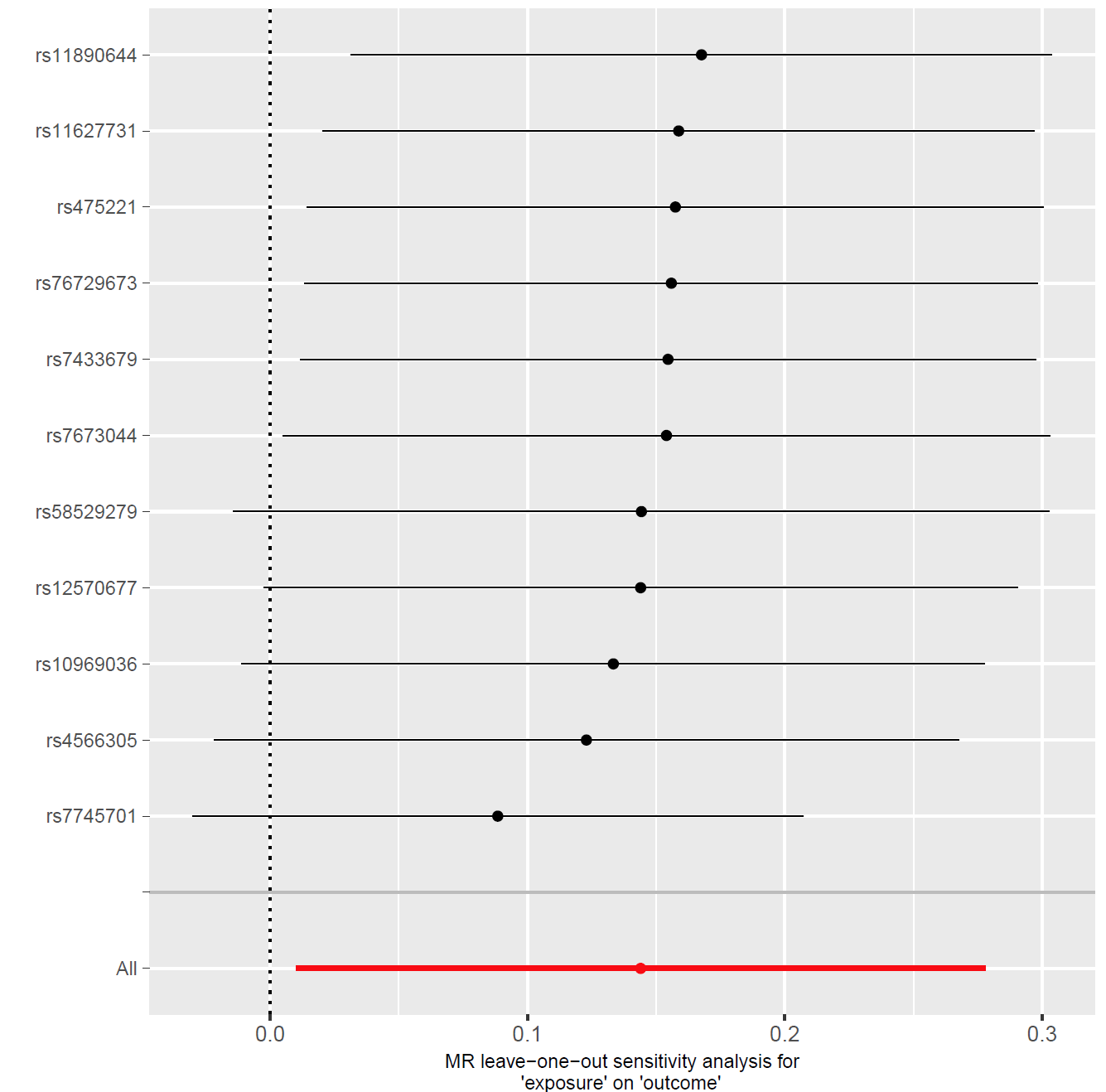

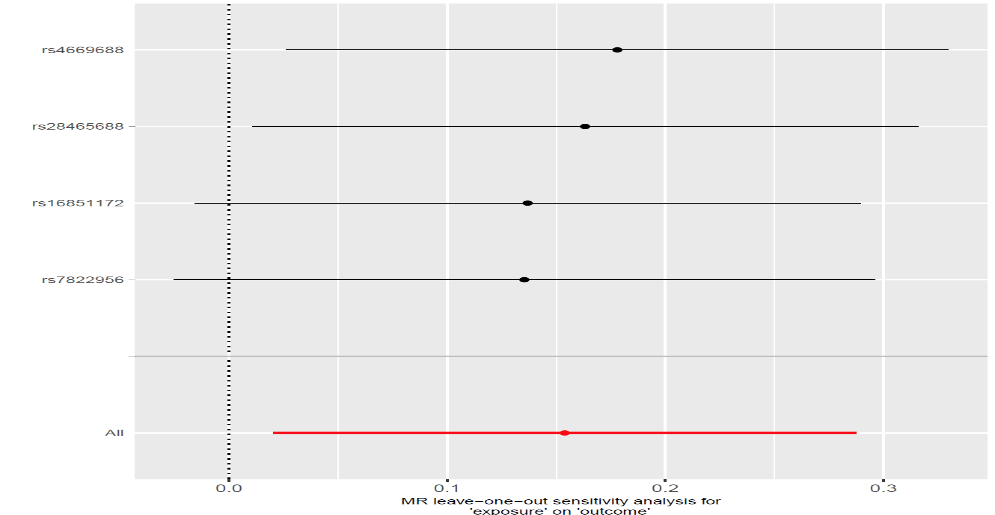


PWY.5791..1.4.dihydroxy.2.naphthoate.biosynthesis.II..plants. PWY.7209..superpathway.of.pyrimidine.ribonucleosides.degradation


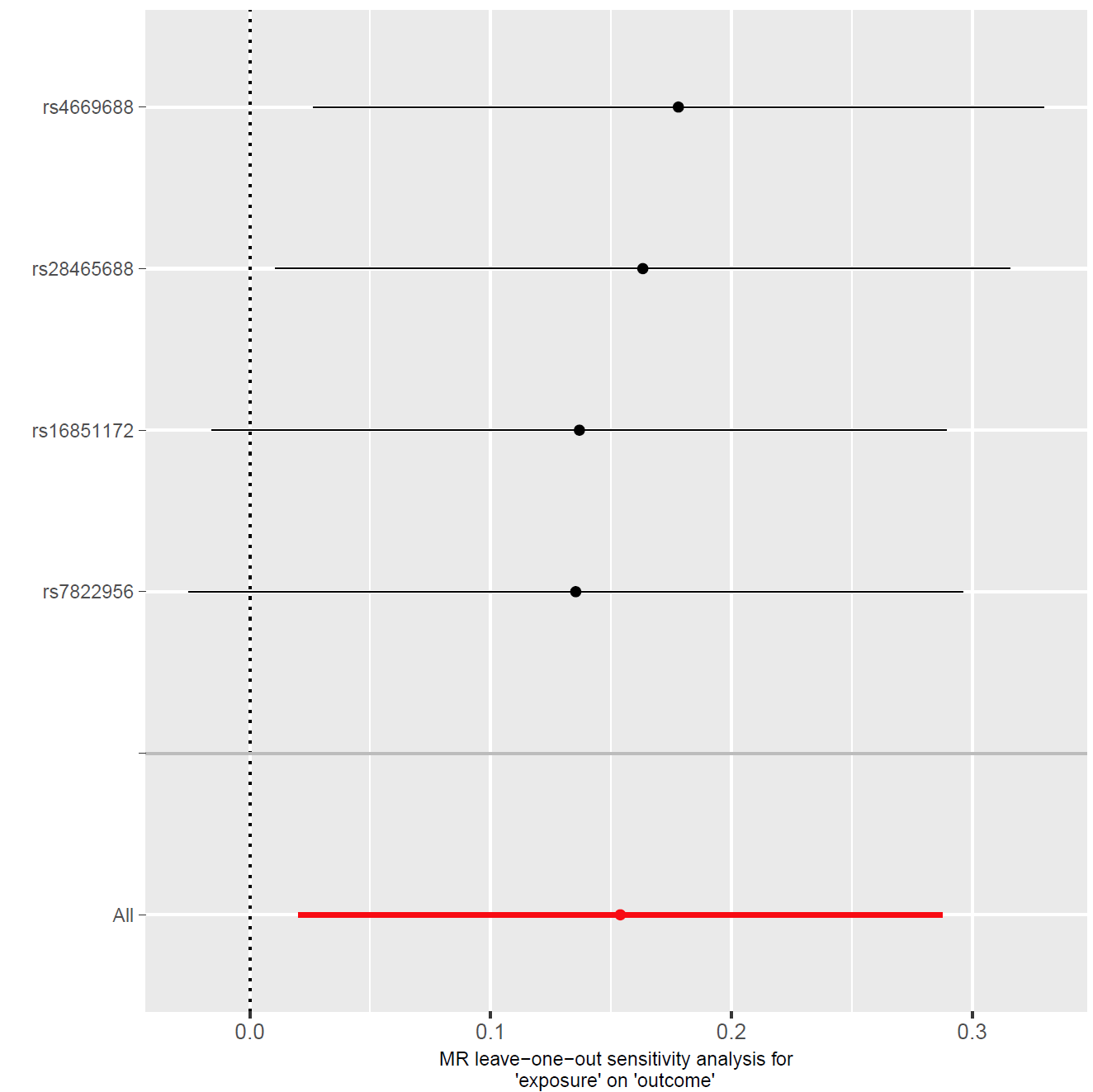

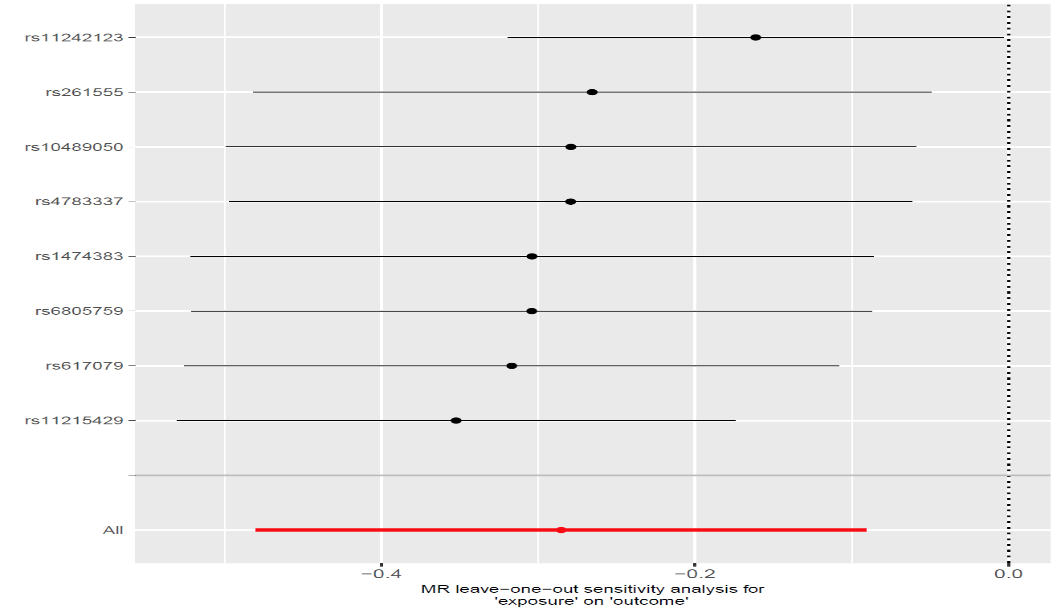


UDPNAGSYN.PWY..UDP.N.acetyl.D.glucosamine.biosynthesis.I k__Bacteria.p__Proteobacteria.c__Betaproteobacteria


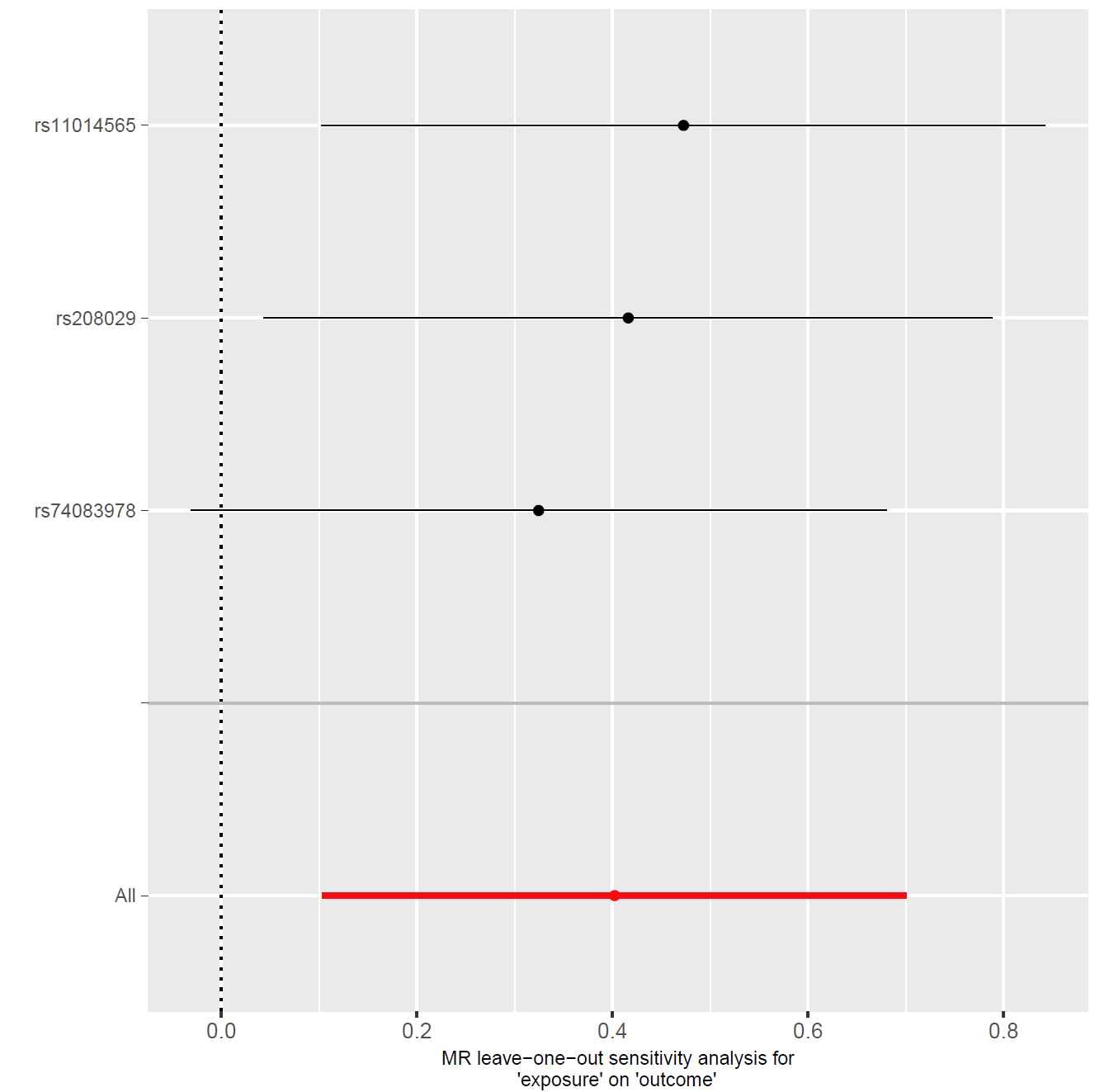

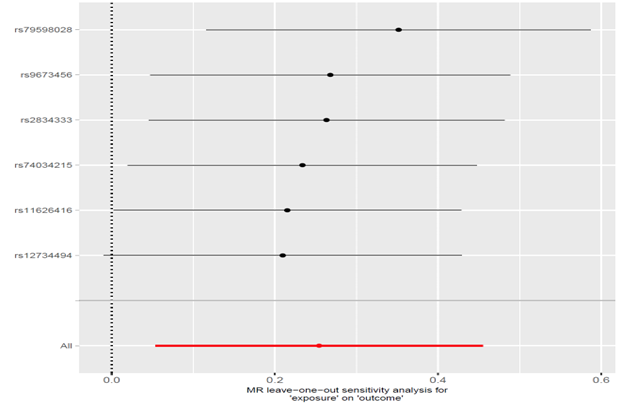


k__Bacteria.p__Firmicutes.c__Bacilli.o__Lactobacillales.f__Lactobacillaceae k__Bacteria.p__Firmicutes.c__Clostridia.o__Clostridiales.f__Clostridiaceae


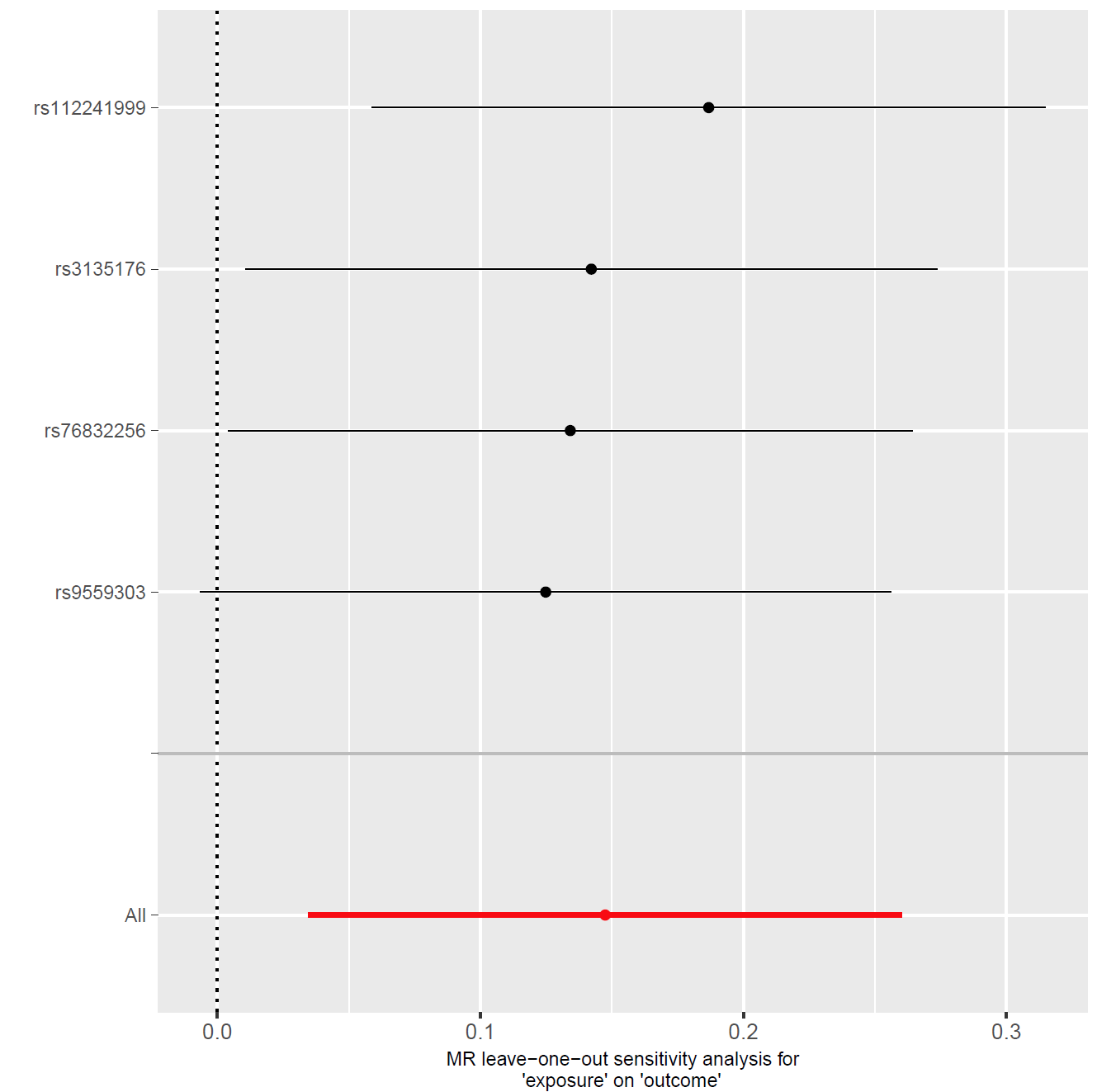

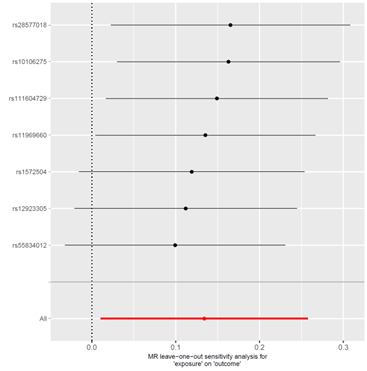


k__Bacteria.p__Firmicutes.c__Clostridia.o__Clostridiales.f__Clostridiaceae.g__Clostridium


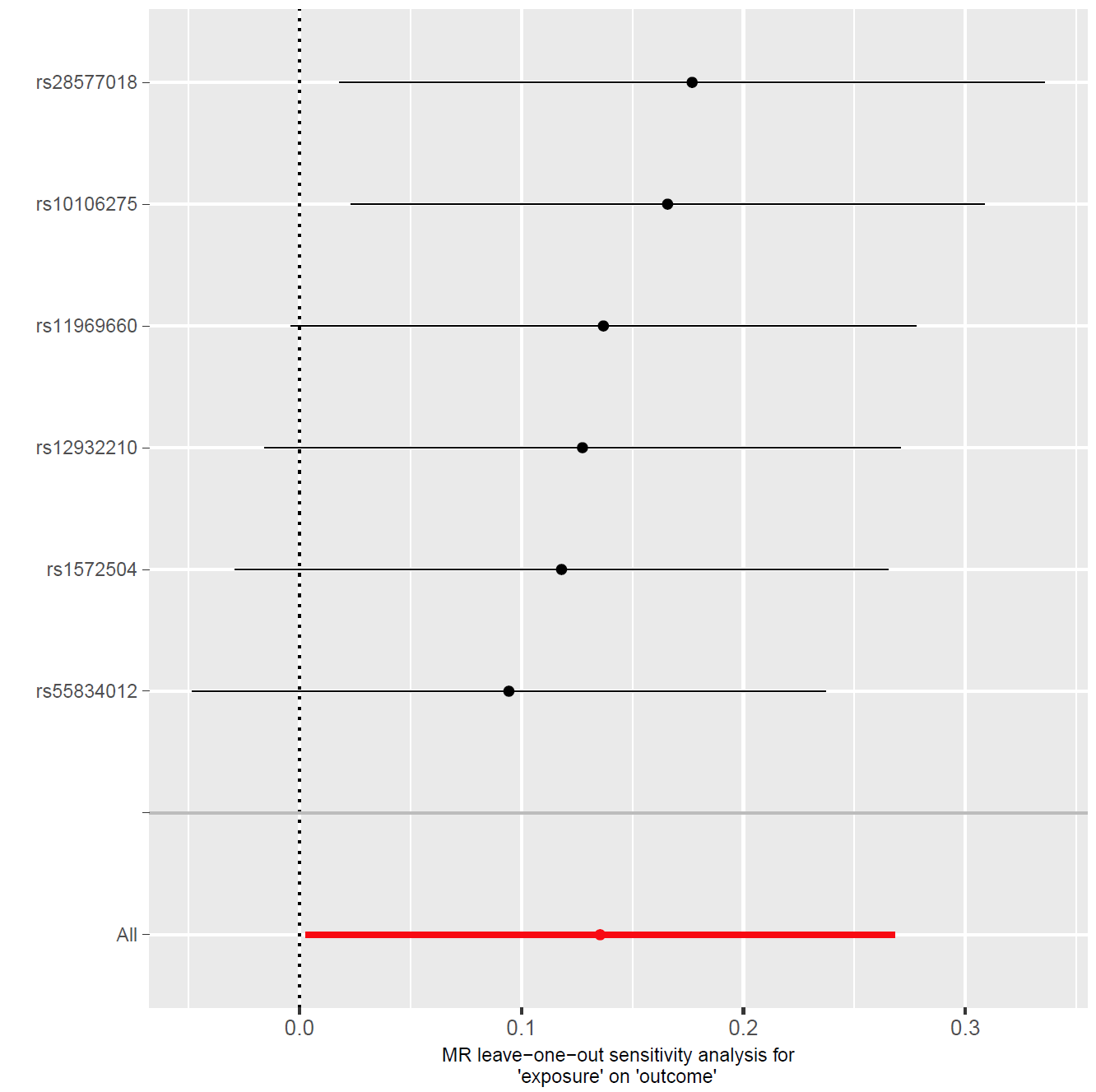


k__Bacteria.p__Proteobacteria.c__Betaproteobacteria.o__Burkholderiales


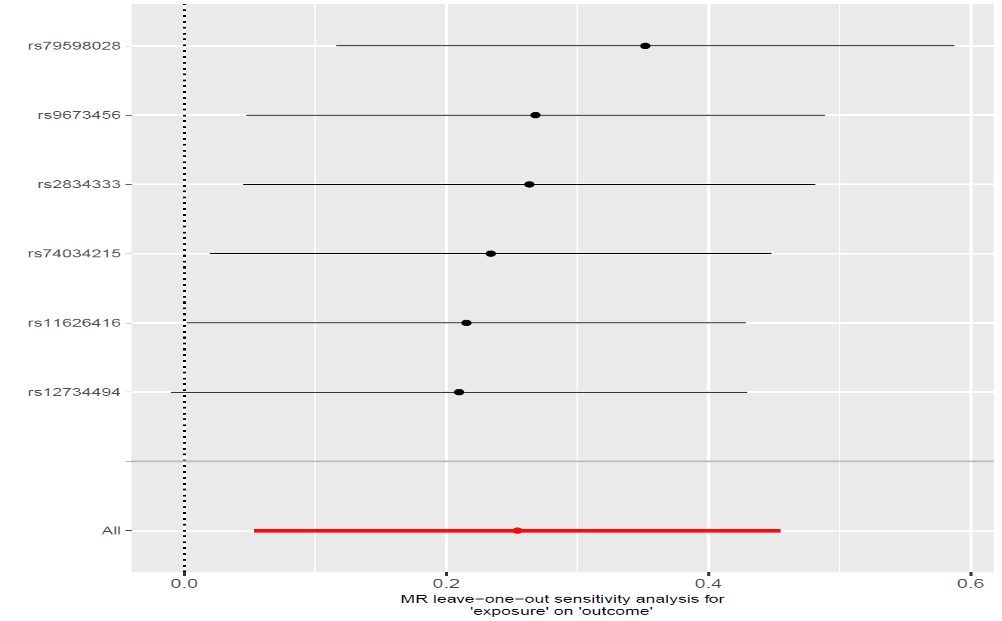


k__Bacteria.p__Bacteroidetes.c__Bacteroidia.o__Bacteroidales.f__Porphyromonadaceae.g__Odoribacter.s__Odoribacter_splanchnicus


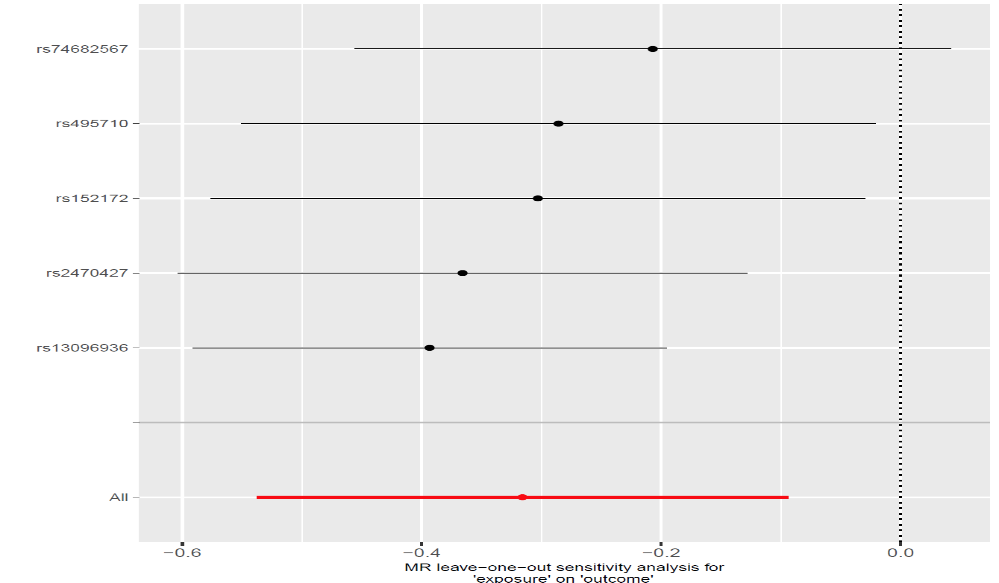


k__Bacteria.p__Firmicutes.c__Clostridia.o__Clostridiales.f__Eubacteriaceae.g__Eubacterium.s__Eubacterium_hallii


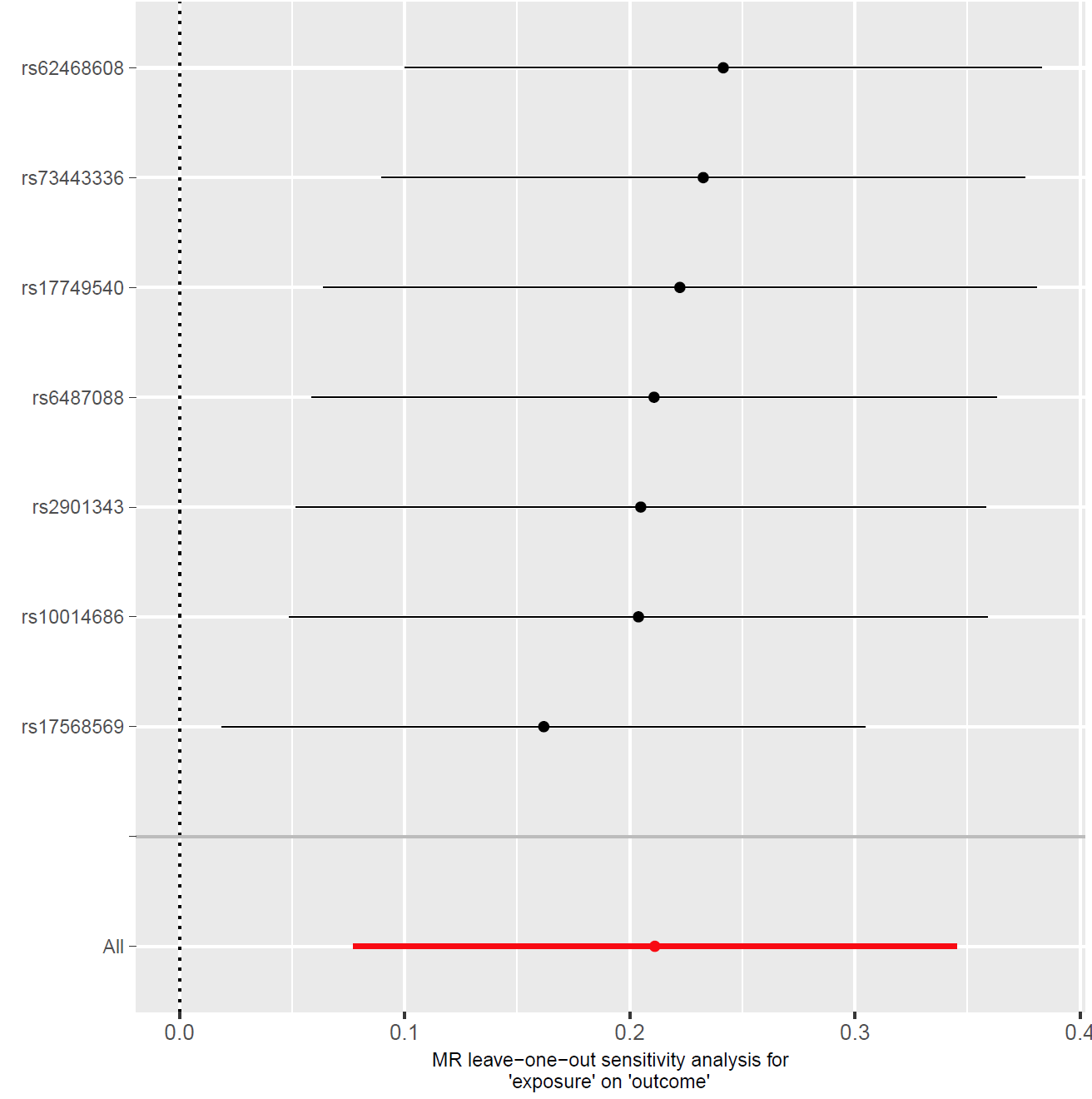


k__Bacteria.p__Firmicutes.c__Clostridia.o__Clostridiales.f__Lachnospiraceae.g__Coprococcus.s__Coprococcus_sp_ART55_1


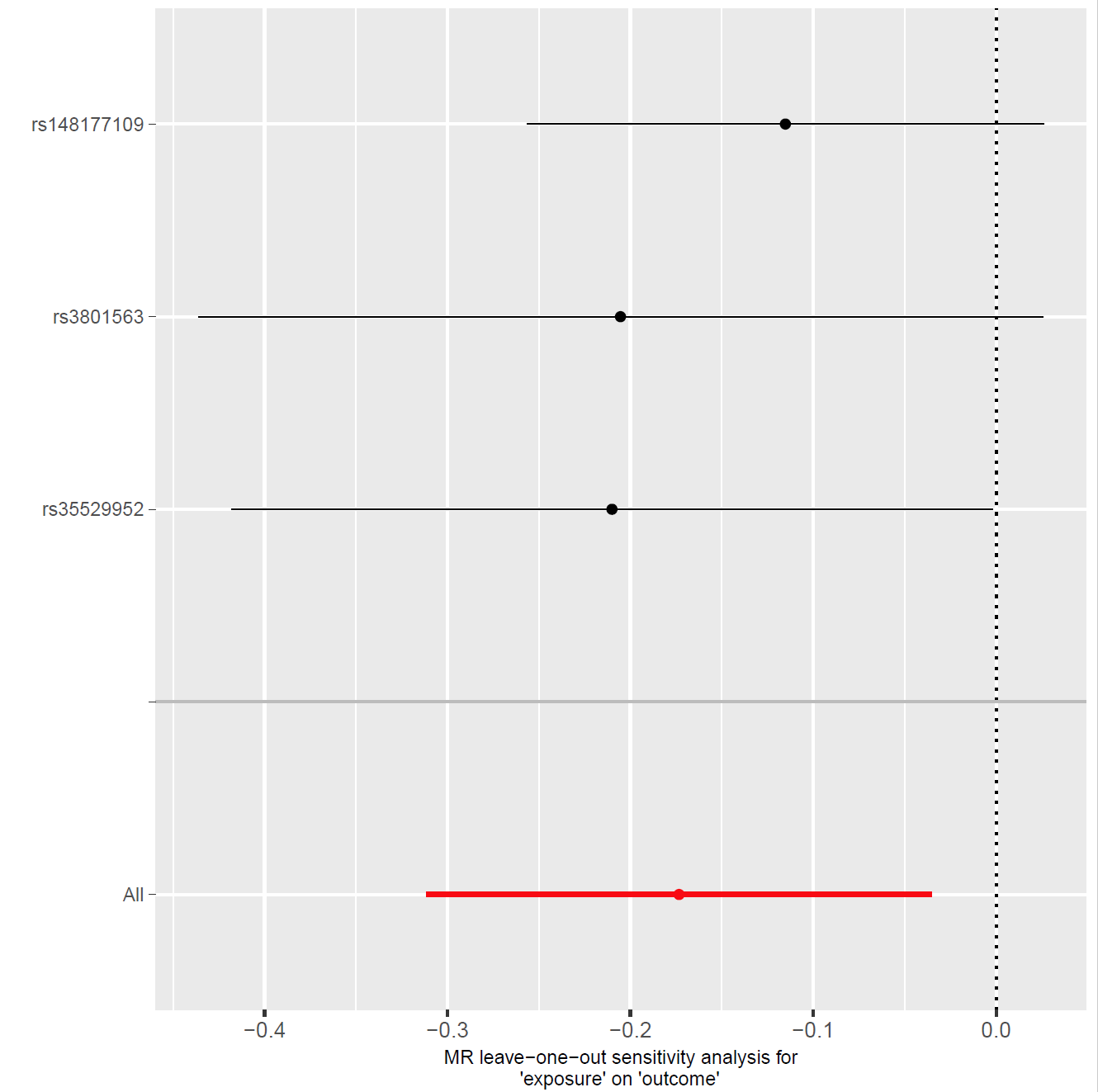


Figure S3. Scatter plots for the causal relationship between gut microbiome and childhood allergy in reverse MR analyses.

NAGLIPASYN.PWY..lipid.IVA.biosynthesis PYRIDNUCSAL.PWY..NAD.salvage.pathway.I


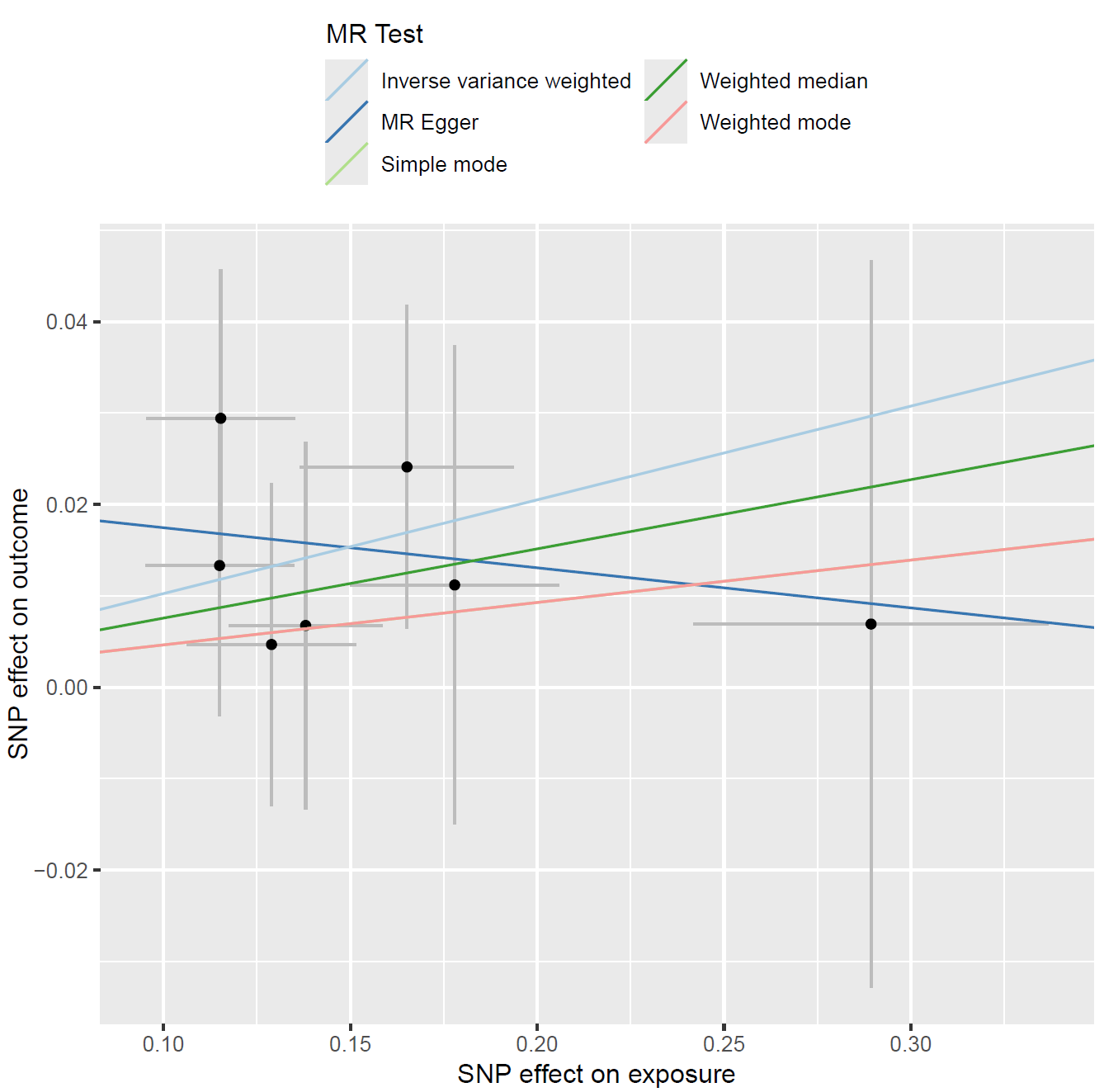

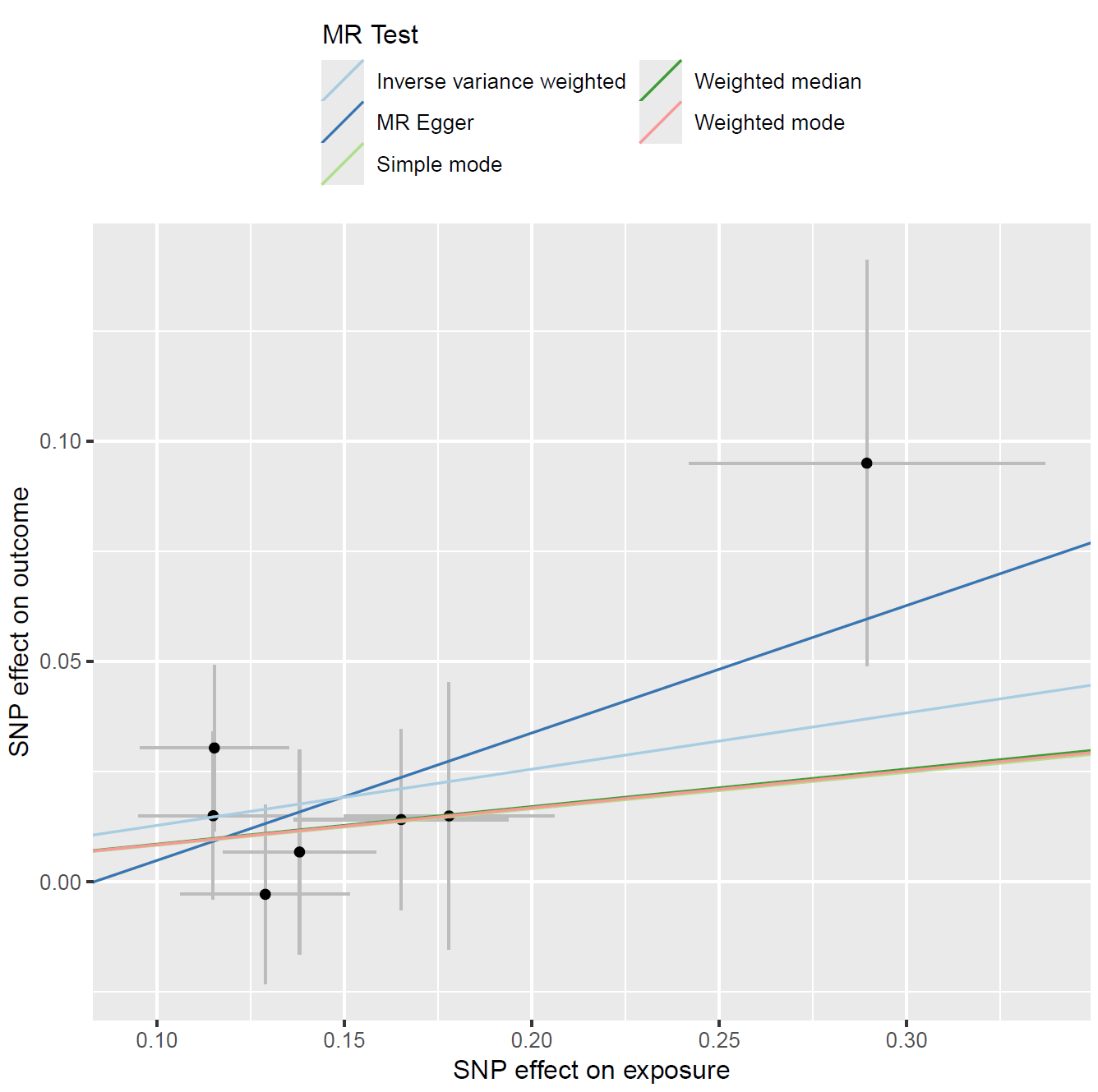


k_Bacteria.p_Bacteroidetes.c_Bacteroidia.o_Bacteroidales.f_Bacteroidaceae


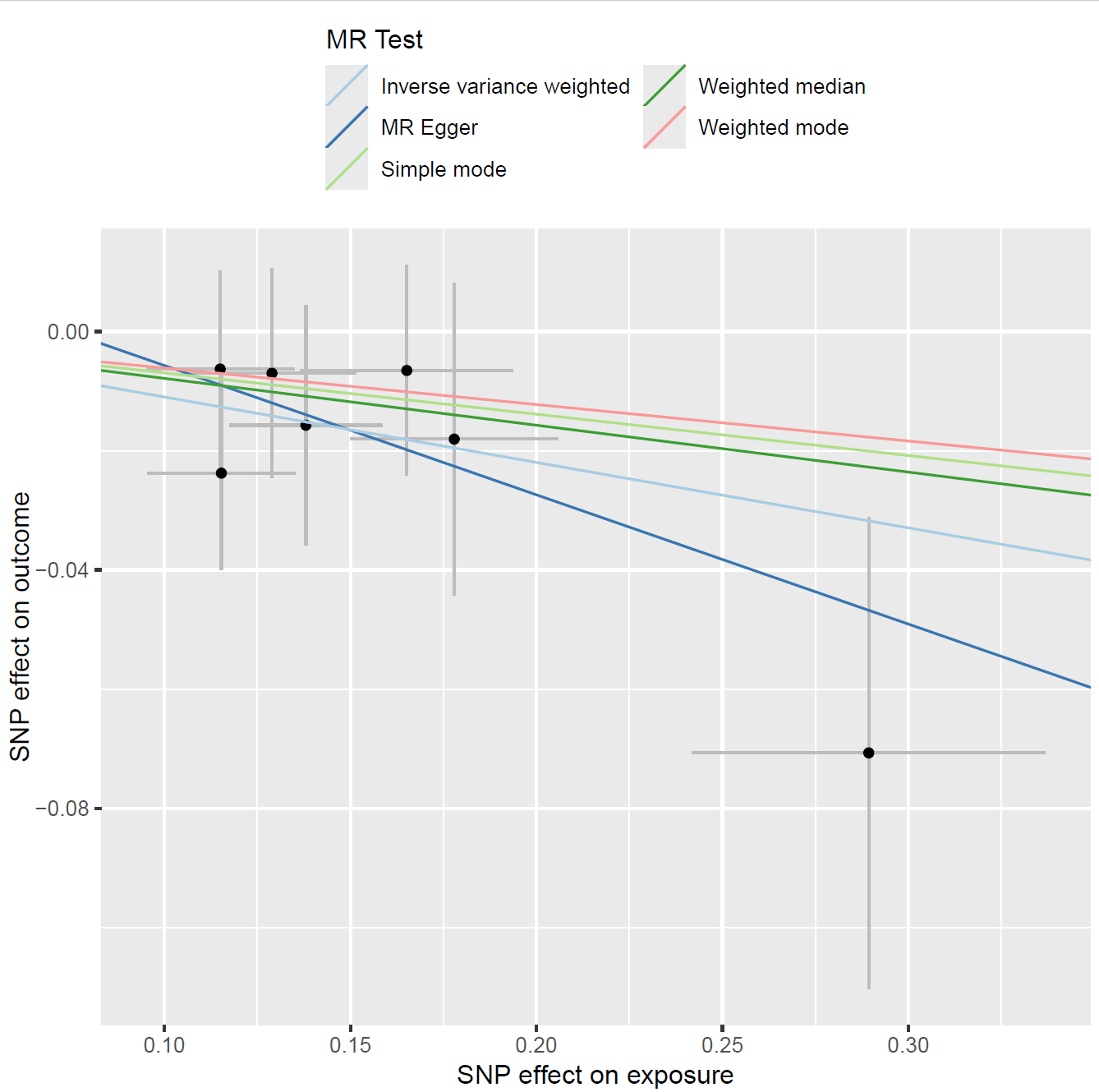


k_Bacteria.p_Actinobacteria.c_Actinobacteria.o_Coriobacteriales.f_Coriobacteriaceae.g_Adlercreutzia


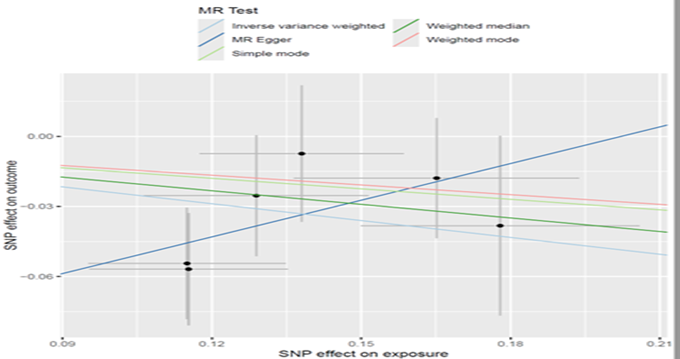


k_Bacteria.p_Bacteroidetes.c_Bacteroidia.o_Bacteroidales.f_Bacteroidaceae.g_Bacteroides


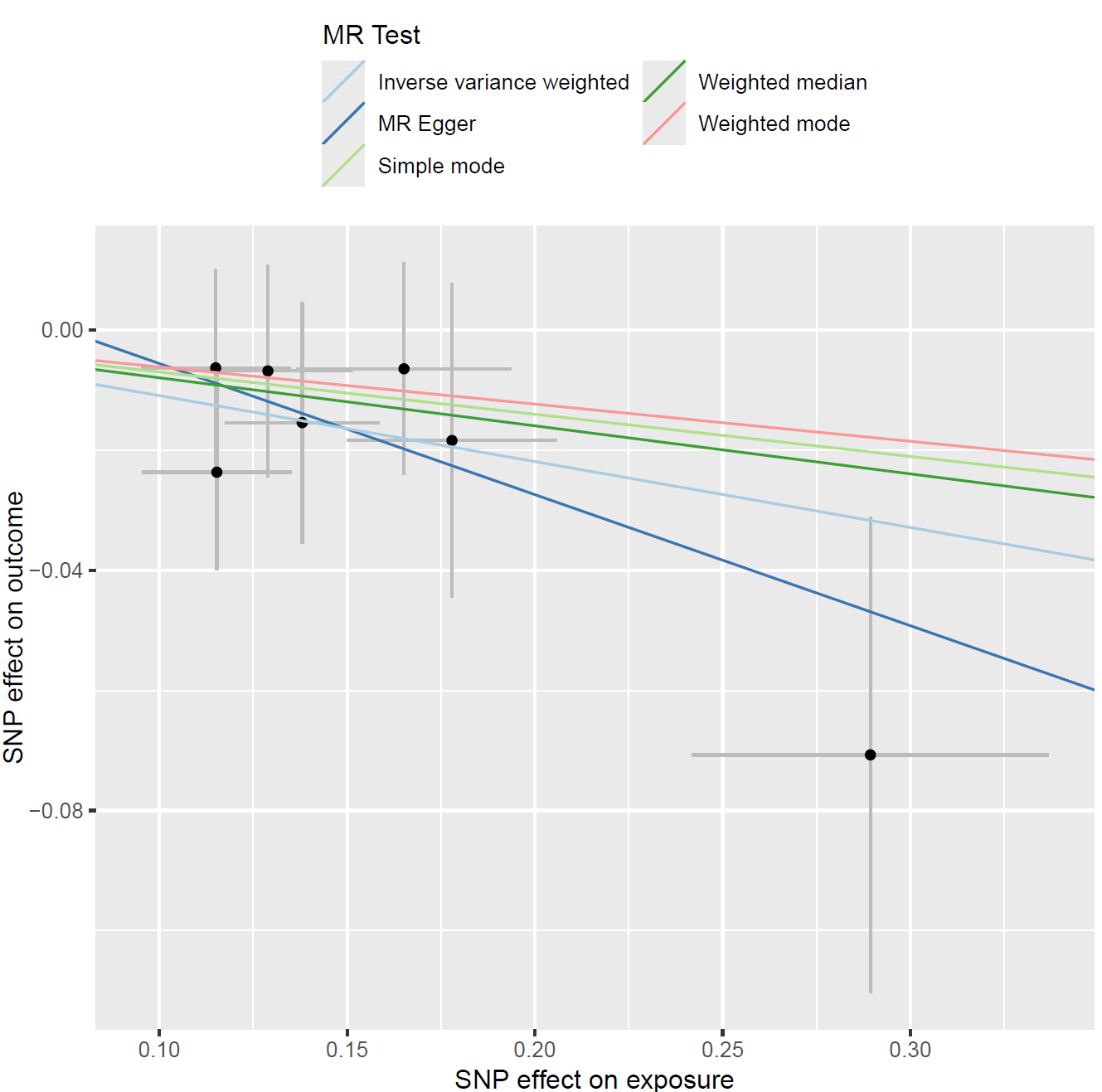


k_Bacteria.p_Firmicutes.c_Clostridia.o_Clostridiales.f_Lachnospiraceae.g_Roseburia


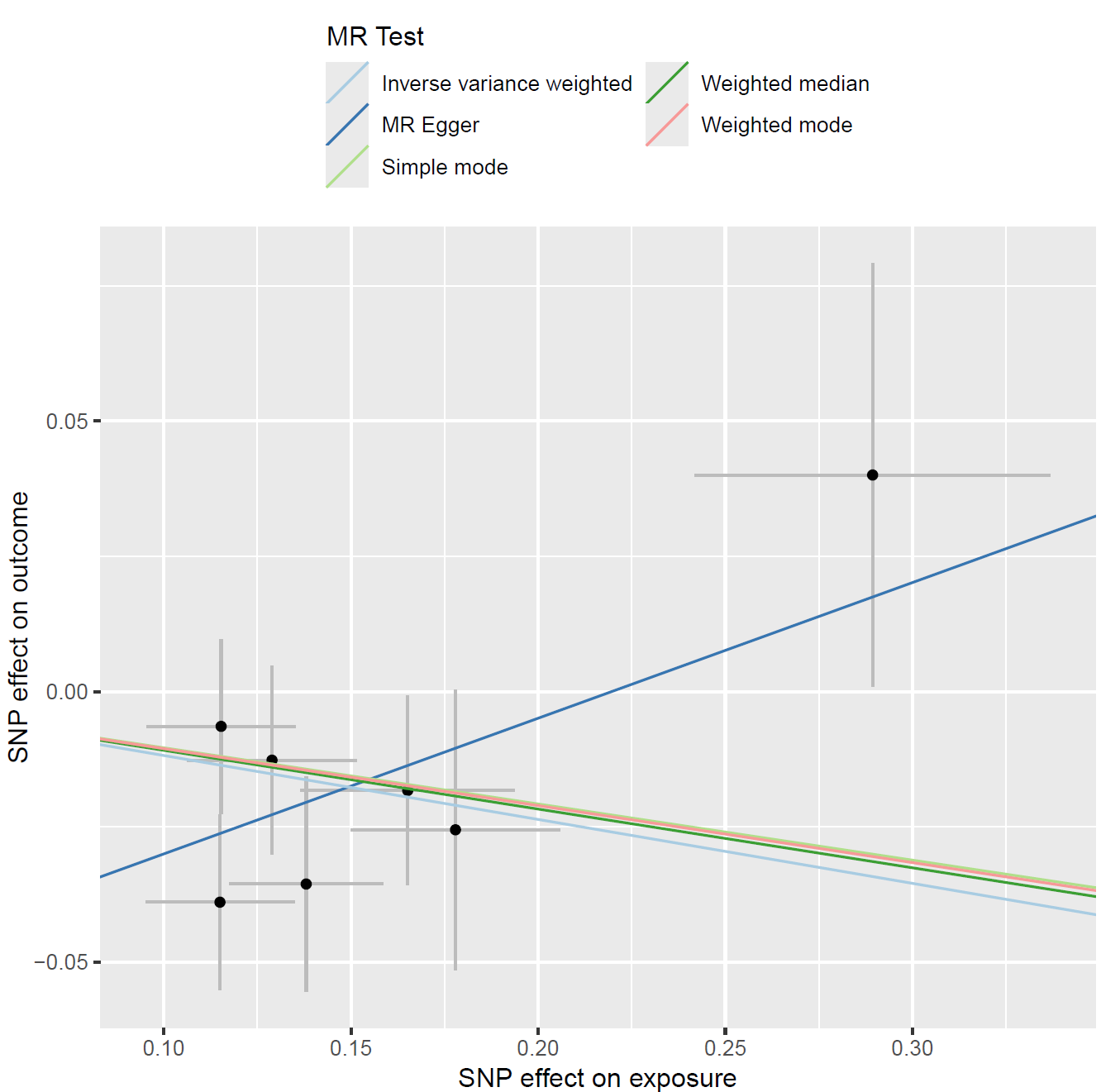


k_Bacteria.p_Actinobacteria.c_Actinobacteria.o_Coriobacteriales.f_Coriobacteriaceae.g_Adlercreutzia.s_Adlercreutzia_equolifaciens


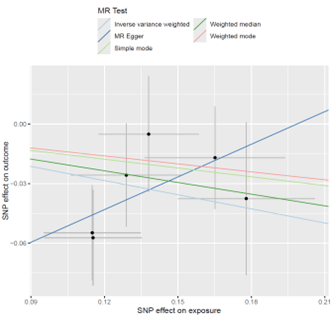


k_Bacteria.p_Firmicutes.c_Erysipelotrichia.o_Erysipelotrichales.f_Erysipelotrichaceae.g_Holdemania.s_Holdemania_unclassified


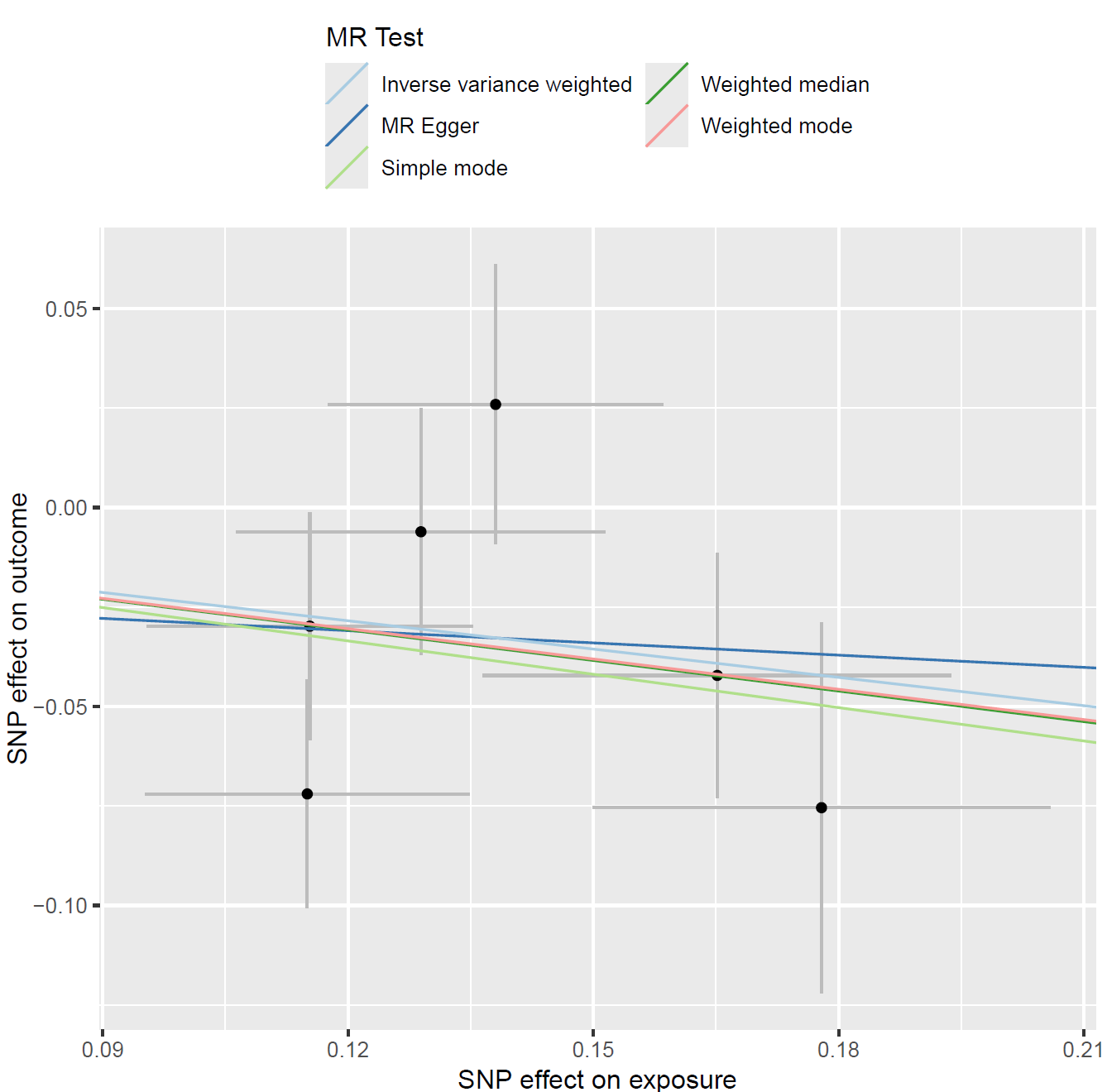


k_Bacteria.p_Bacteroidetes.c_Bacteroidia.o_Bacteroidales.f_Bacteroidaceae.g_Bacteroides.s_Bacteroides_vulgatus


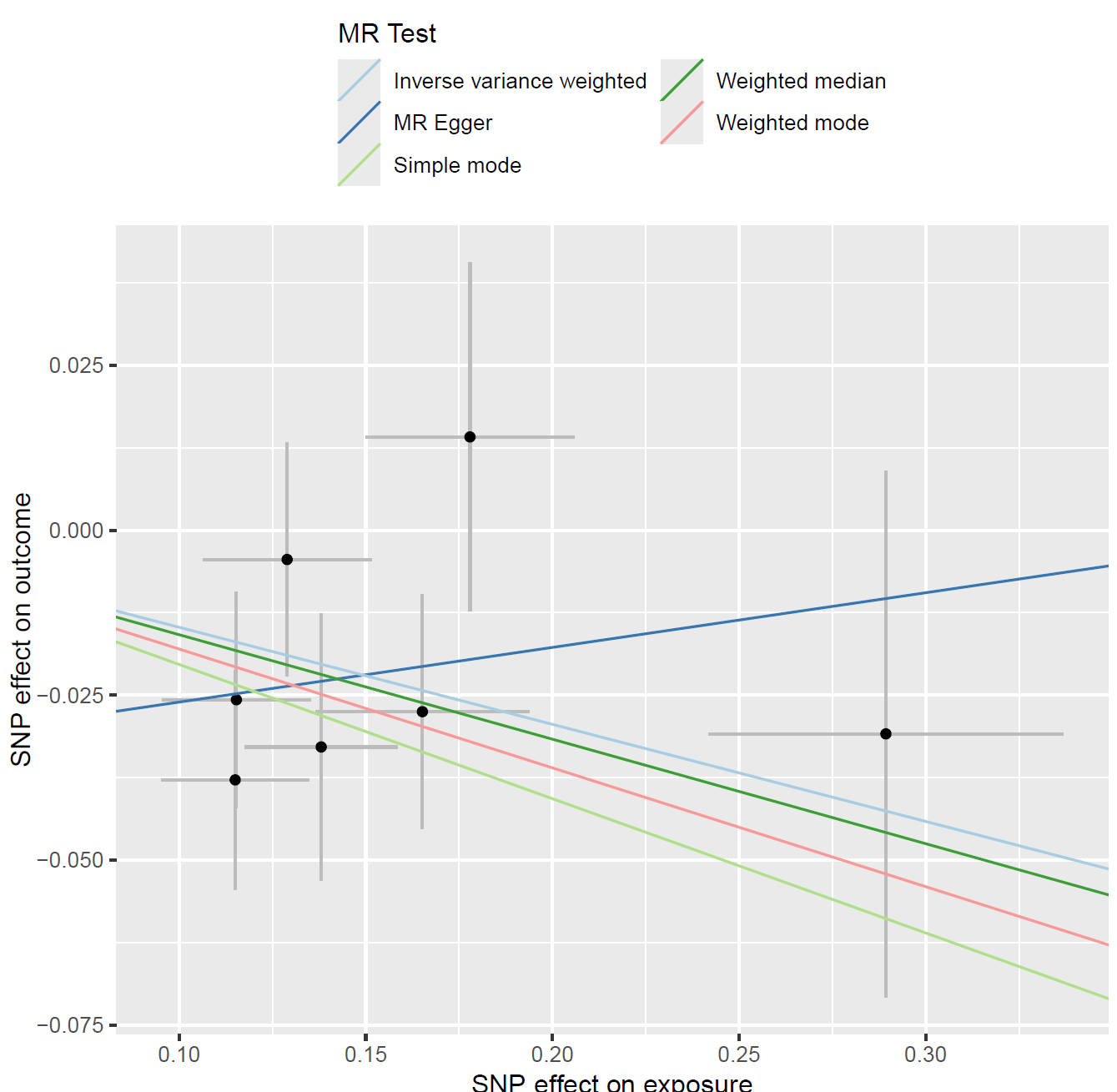


Figure S4. Leave-one-out plots for the causal relationship between gut microbiome and childhood allergy in reverse MR analyses.

NAGLIPASYN.PWY..lipid.IVA.biosynthesis PYRIDNUCSAL.PWY..NAD.salvage.pathway.I


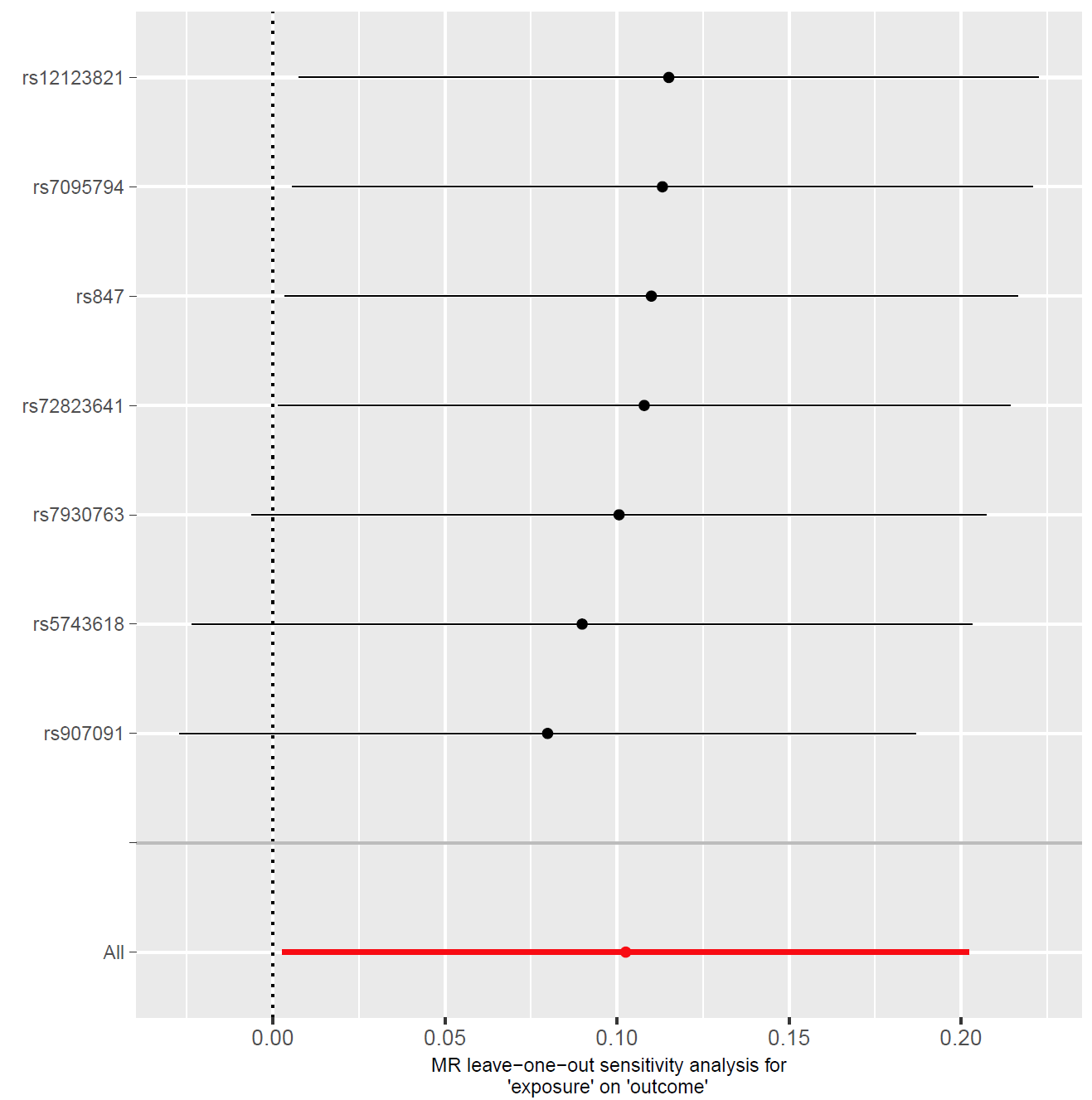

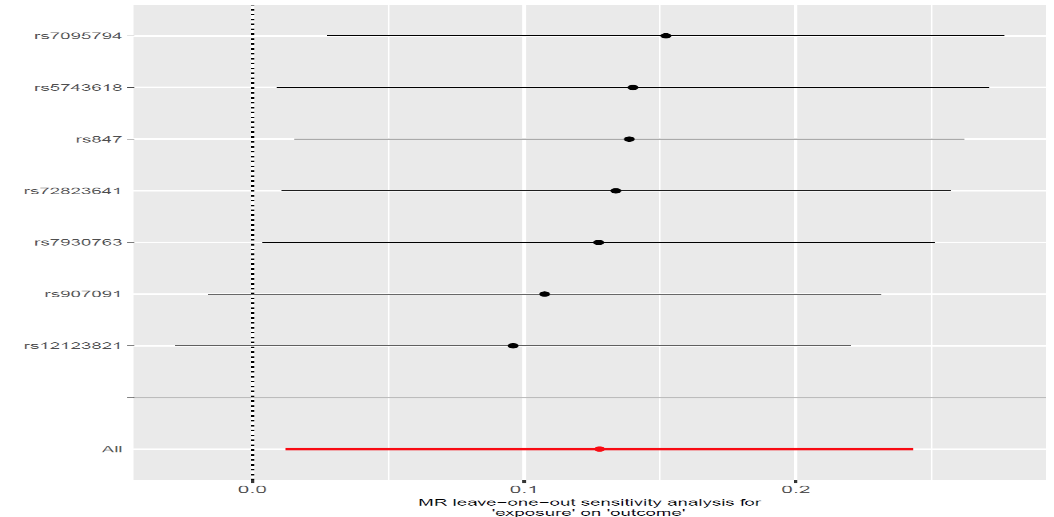


k_Bacteria.p_Bacteroidetes.c_Bacteroidia.o_Bacteroidales.f_Bacteroidaceae


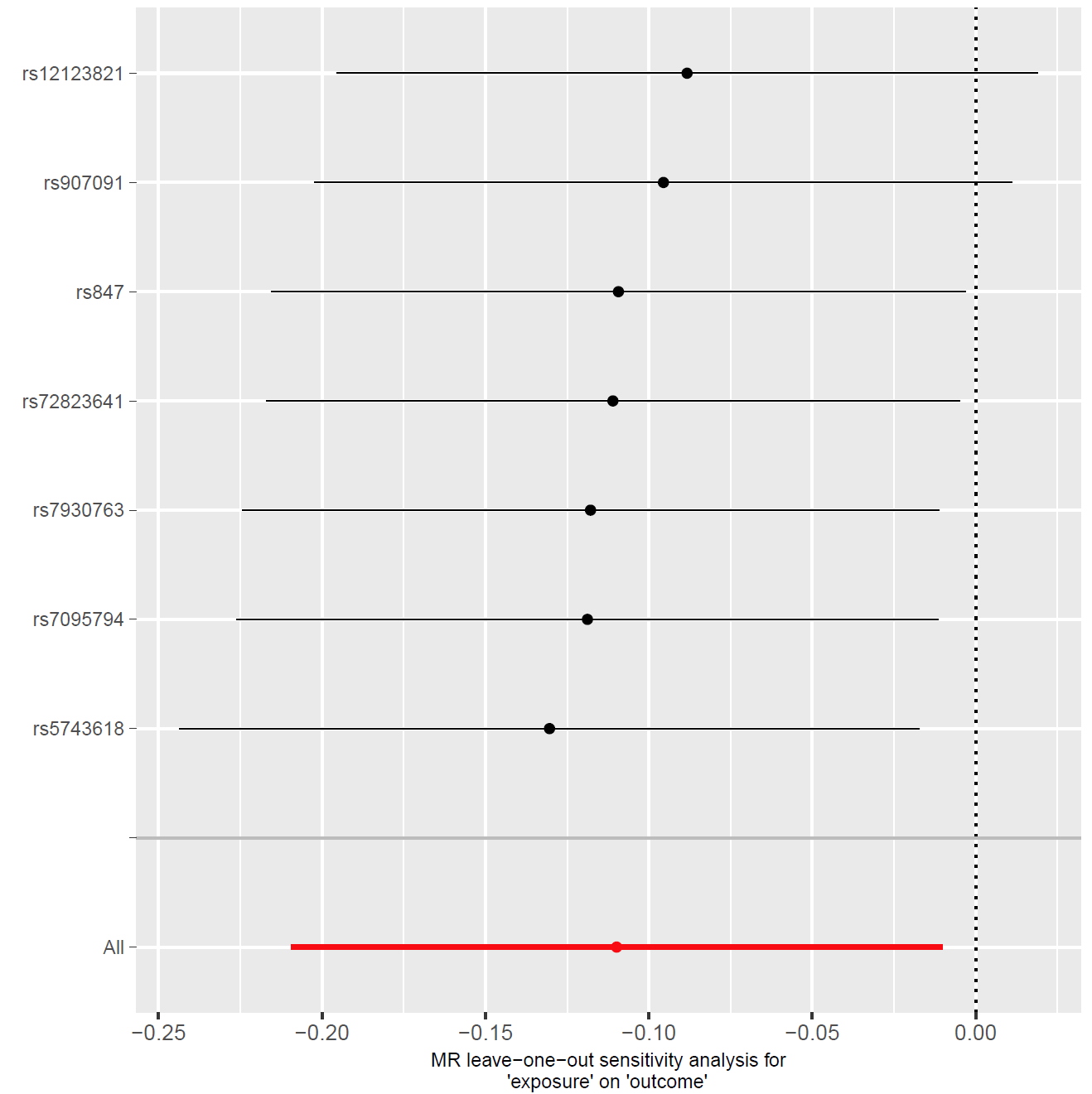


k_Bacteria.p_Actinobacteria.c_Actinobacteria.o_Coriobacteriales.f_Coriobacteriaceae.g_Adlercreutzia


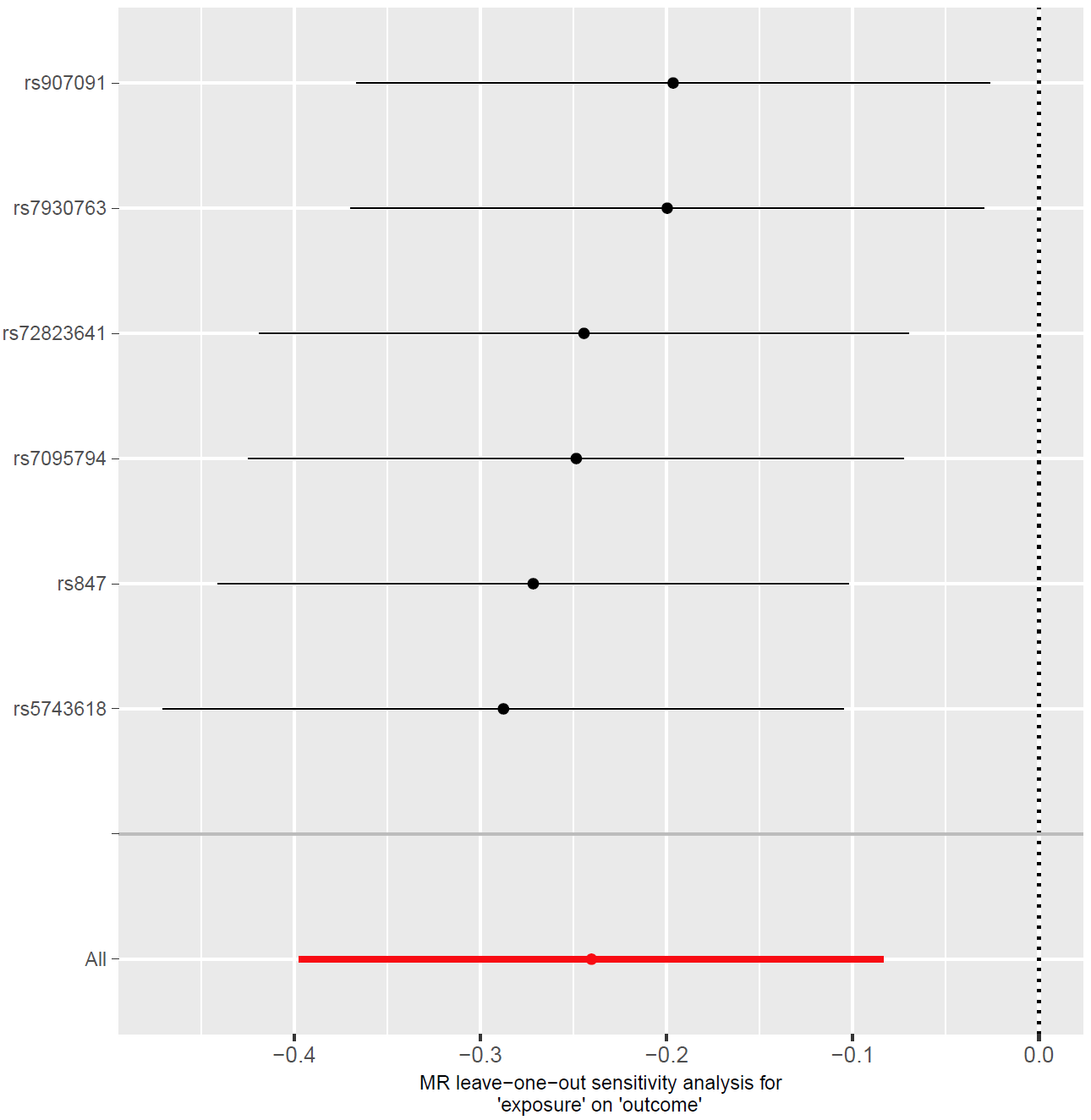


k_Bacteria.p_Bacteroidetes.c_Bacteroidia.o_Bacteroidales.f_Bacteroidaceae.g_Bacteroides


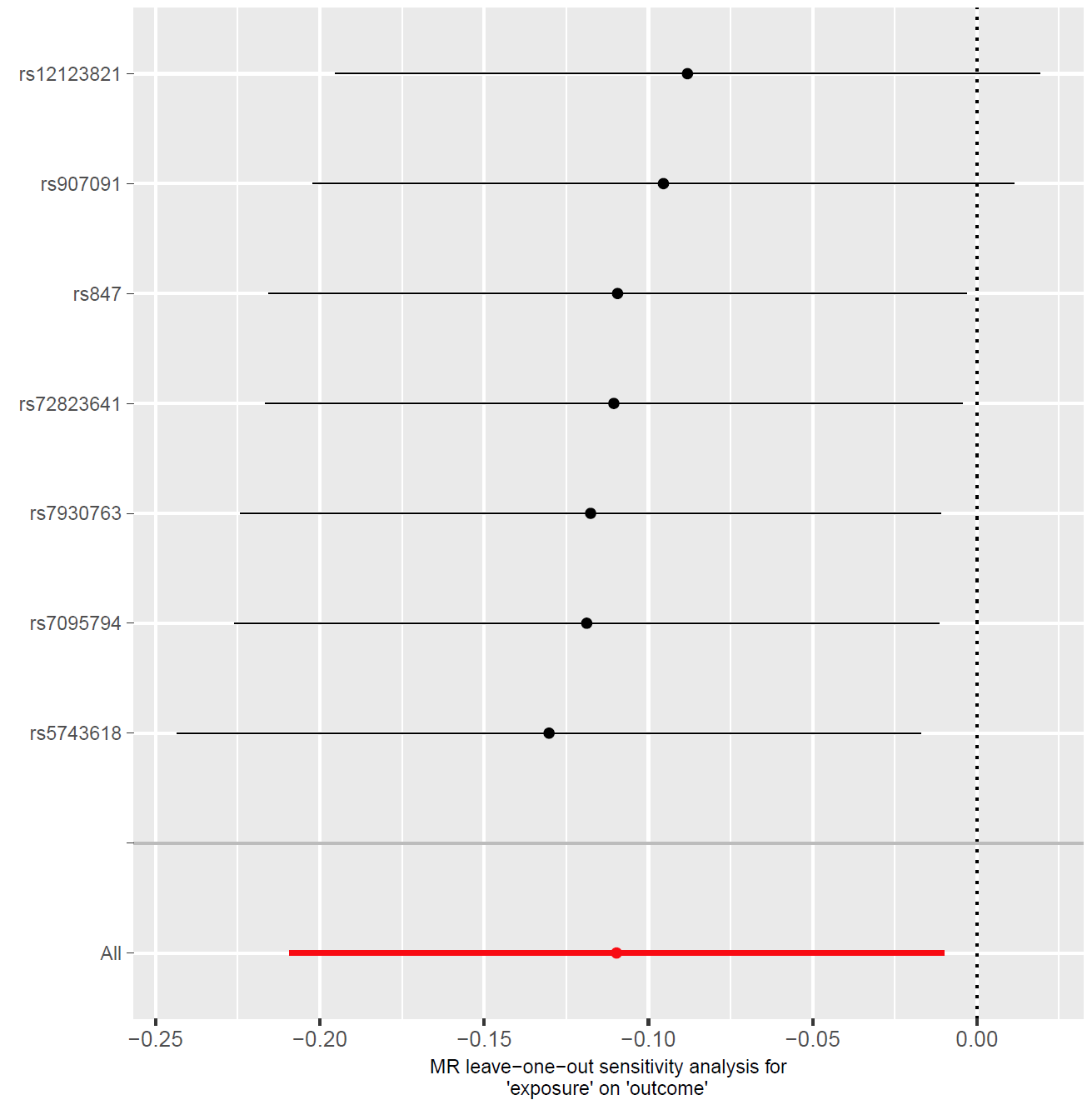


k_Bacteria.p_Firmicutes.c_Clostridia.o_Clostridiales.f_Lachnospiraceae.g_Roseburia


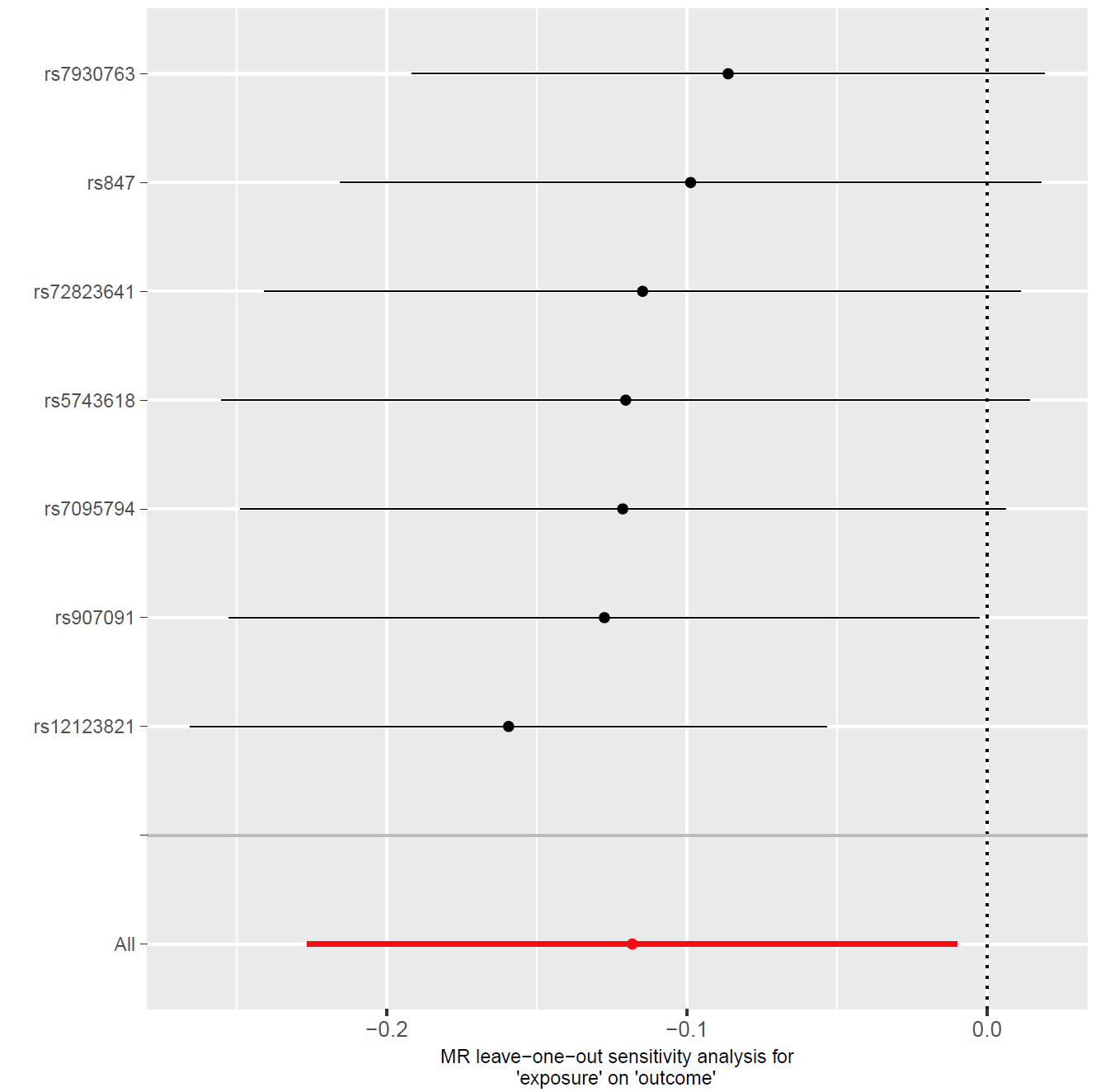


k_Bacteria.p_Actinobacteria.c_Actinobacteria.o_Coriobacteriales.f_Coriobacteriaceae.g_Adlercreutzia.s_Adlercreutzia_equolifaciens


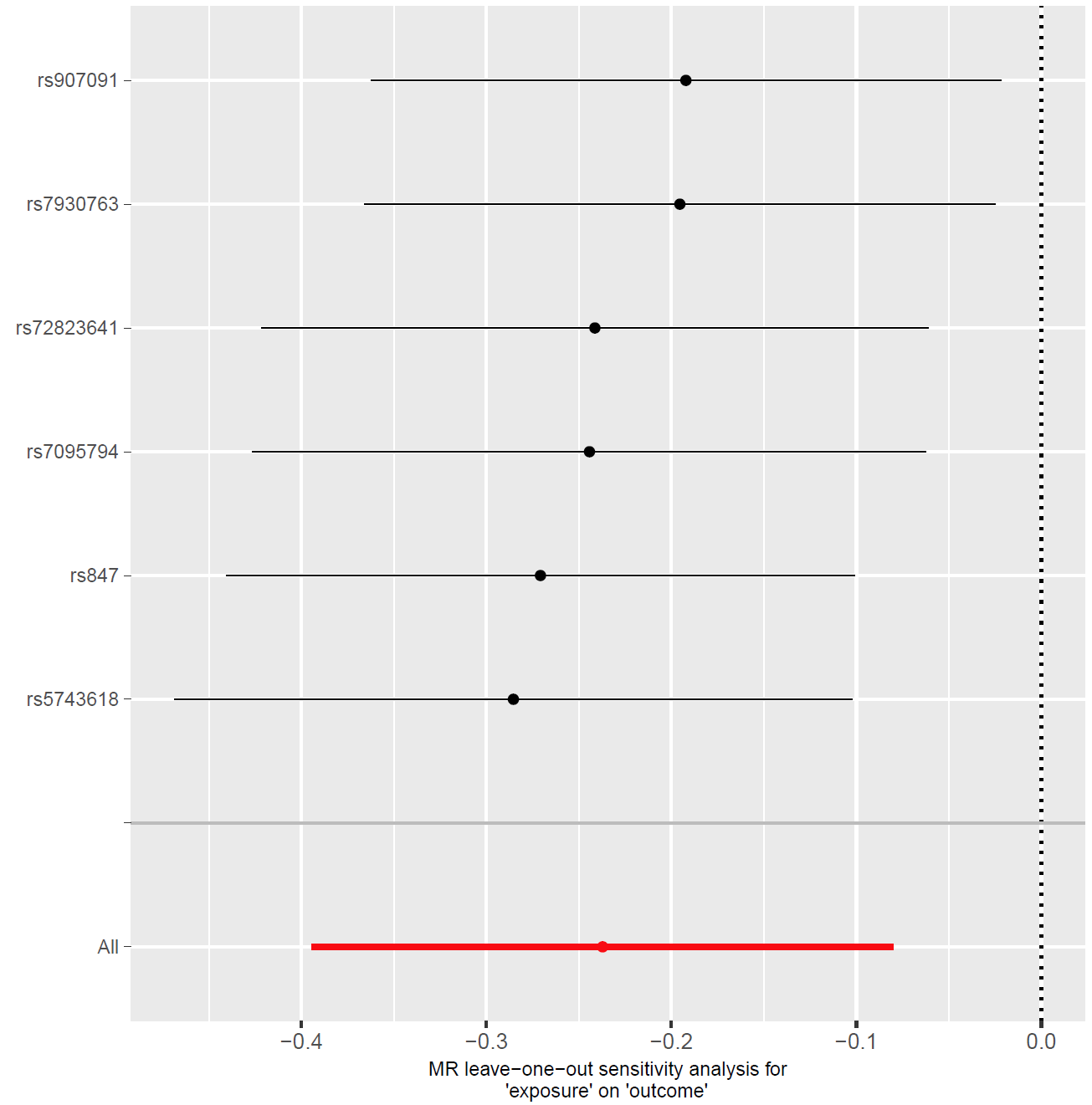


k_Bacteria.p_Firmicutes.c_Erysipelotrichia.o_Erysipelotrichales.f_Erysipelotrichaceae.g_Holdemania.s_Holdemania_unclassified


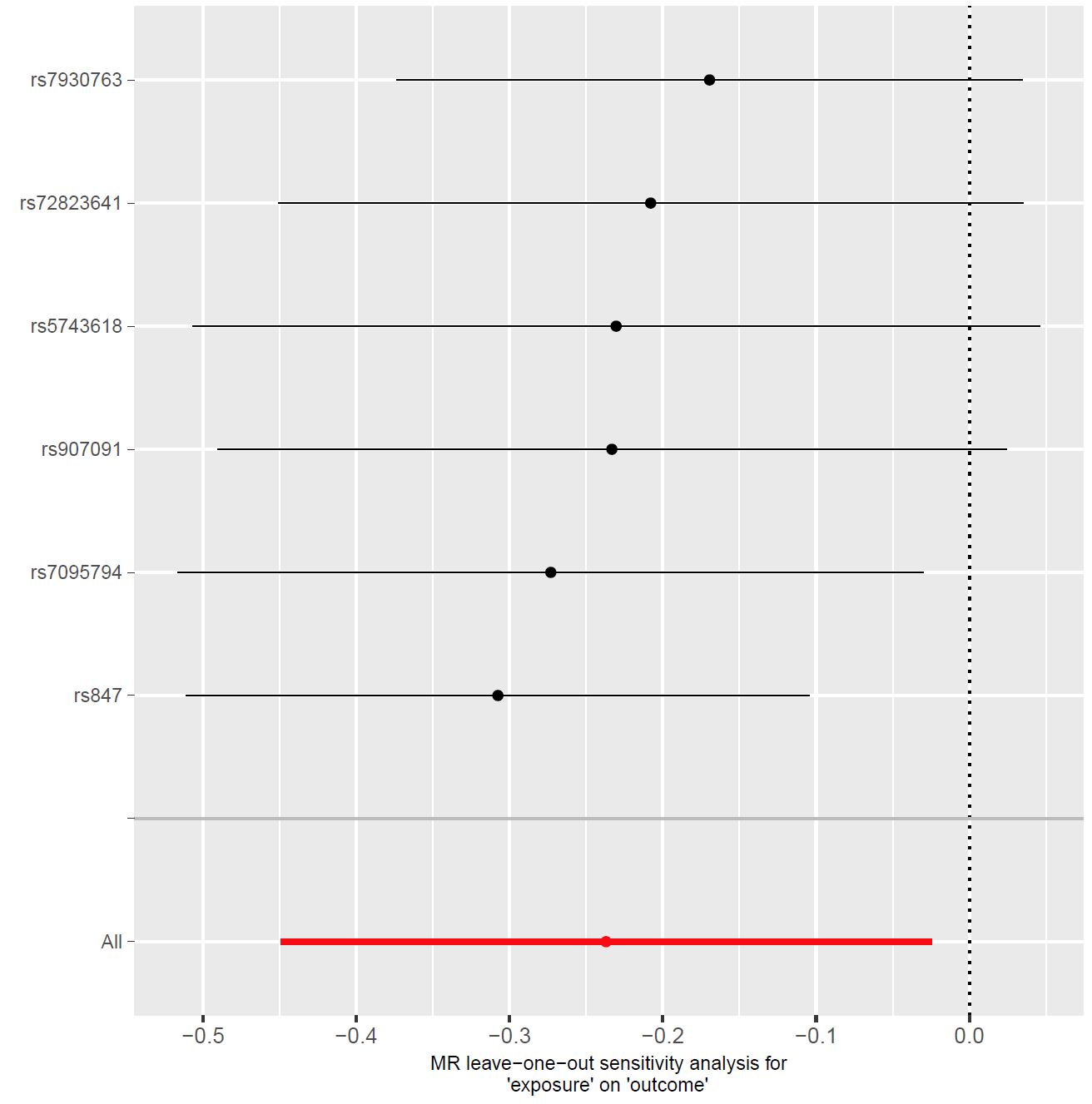


k_Bacteria.p_Bacteroidetes.c_Bacteroidia.o_Bacteroidales.f_Bacteroidaceae.g_Bacteroides.s_Bacteroides_vulgatus


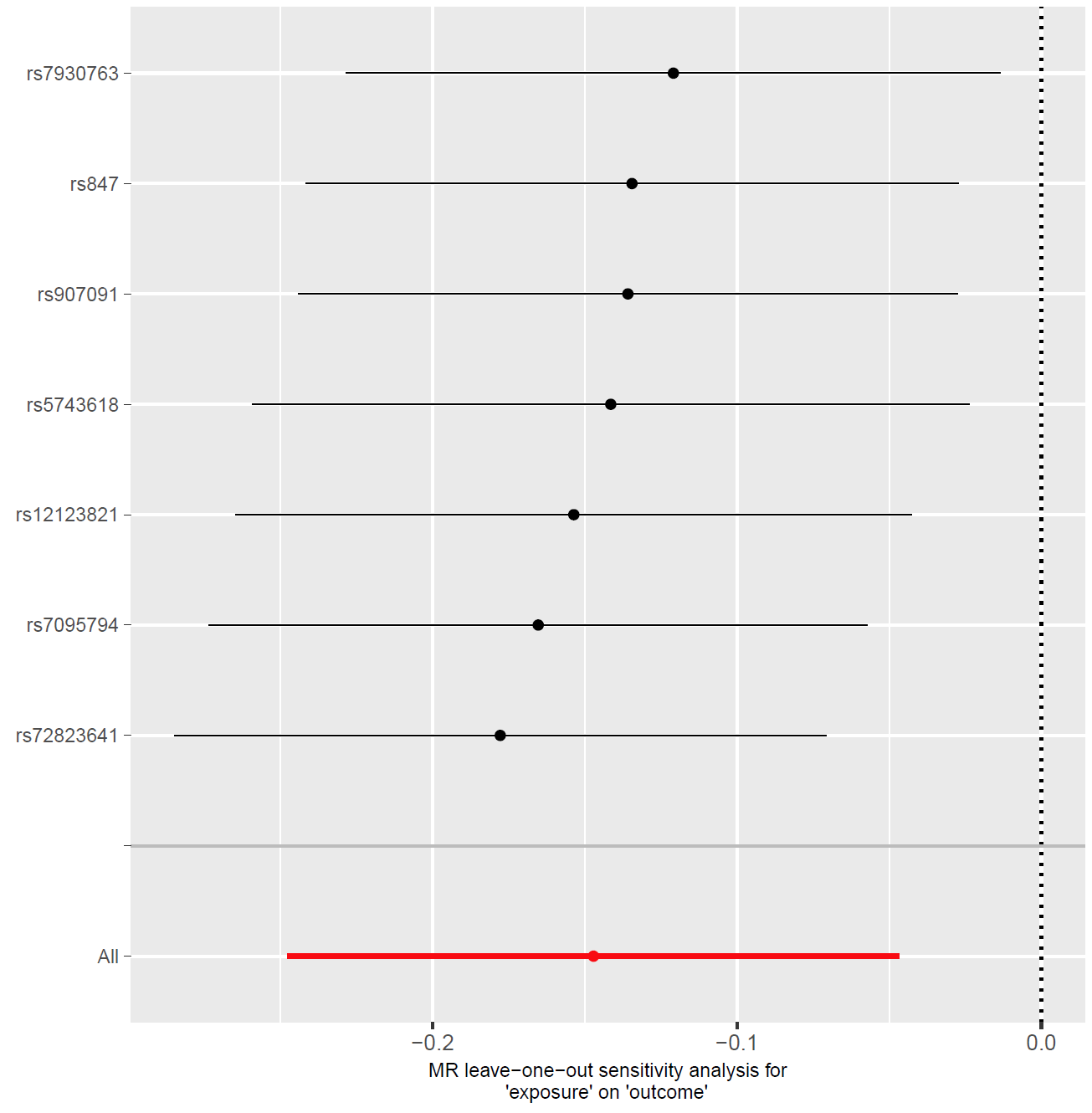

Supplement: Supplementary file 2 [file medi-105-e47793-s002.doc]
